# *n*‑Mode Quantized Anharmonic Vibronic Hamiltonians for Matrix Product State Dynamics

**DOI:** 10.1021/acs.jctc.5c02014

**Published:** 2026-02-12

**Authors:** Valentin Barandun, Nina Glaser, Markus Reiher

**Affiliations:** † Department of Chemistry and Applied Biosciences, ETH Zürich, Vladimir-Prelog-Weg 2, 8093 Zürich, Switzerland; ‡ NNF Quantum Computing Programme, Niels Bohr Institute, University of Copenhagen, Blegdamsvej 21, København Ø, 2100 Copenhagen, Denmark

## Abstract

Theoretical predictions of photochemical processes are essential for interpreting and understanding spectral features. Reliable quantum dynamics calculations of vibronic systems require precise modeling of anharmonic effects in the potential energy surfaces and off-diagonal nonadiabatic coupling terms. In this work, we present the *n*-mode quantization of all vibronic Hamiltonian terms comprised of general high-dimensional model representations. We expand the existing vibrational DMRG formalism by applying the *n*-mode quantization to all potential energy surfaces entering the Hamiltonian as well as to all off-diagonal coupling terms. This is accompanied by the introduction of a novel matrix product state architecture employing tailored local site operators, which allow an effective encoding of the vibronic wave function in a tensor-train format. This results in a second-quantized framework for accurate vibronic calculations employing the density matrix renormalization group algorithm. We demonstrate the accuracy and reliability of this approach by calculating the excited-state quantum dynamics of maleimide. We analyze convergence and the choice of parameters of the underlying time-dependent density matrix renormalization group algorithm for the *n*-mode vibronic Hamiltonian, demonstrating that it enables accurate calculations of complex photochemical dynamics.

## Graphical abstract

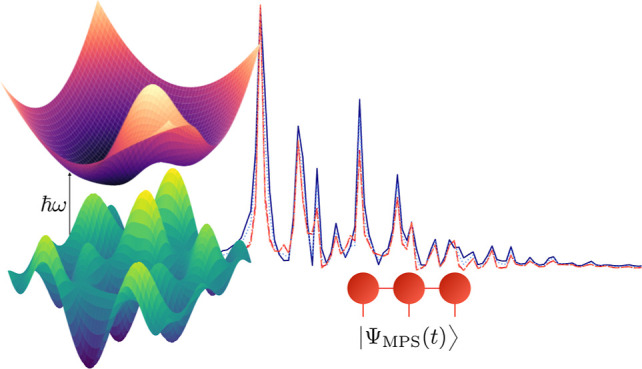

## Introduction

Spectroscopy of vibrationally resolved electronic transitions is a powerful instrument for probing the structure, dynamics, and properties of molecular systems.^1–3^ Their interpretation necessitates reliable theoretical approaches capable of elucidating and predicting spectral features. A central challenge in this endeavor is the accurate modeling of the vibronic Hamiltonian that governs optical phenomena, which must capture all relevant interactions and their functional dependencies.^4–6^ This includes an accurate description of the potential energy surfaces (PES) of the electronic states that enter the vibronic Hamiltonian. Often, approximating the PES as a quadratic function, known as the harmonic approximation, is insufficient to properly describe the vibrational motion of nuclei.^7,8^ Additionally, the nonadiabatic coupling terms that appear in the off-diagonal blocks of the vibronic Hamiltonian govern photochemical and photobiological processes.^9,10^ These nonadiabatic coupling terms can be of complex functional form, and therefore, only a nonrestrictive approach should allow for an accurate description of these terms.

Both the anharmonicity in the PES and the functional form of the nonadiabatic coupling terms can be described by the *n*-mode expansion, a many-body expansion allowing the description of the Hamiltonian terms with a high degree of accuracy.^11^ The idea of the *n*-mode representation for vibrational Hamiltonians was introduced by Jung and Gerber in 1996, who provided expressions up to two-body interactions in the vibrational coordinates, and by Bowman and co-workers, who gave expressions up to fourth order.^12,13^ It offers a convenient way to formulate second-quantized bosonic Hamiltonians.^11^ This second-quantized formulation is a necessity when integrating this approach with some tensor-network methods, which have shown exceptional results in terms of accuracy and computational cost.^14^

One of the most commonly employed tensor-network algorithms is the density matrix renormalization group (DMRG), which parametrizes the wave function as a tensor-train, also referred to as a matrix product state (MPS), which allows for variational optimizations of bosonic and fermionic wave functions, while scaling polynomially in system size.^15–18^

In the MPS parametrization of the wave function, a single tensor is defined per lattice site, which corresponds to a physical degree of freedom such as an orbital or a (vibrational) modal, connected by contracting indices. The core idea of DMRG is partitioning the optimization problem of diagonalizing the Hamiltonian into a series of many smaller eigenvalue problems. Thereby, a single site of an MPS is optimized at a time, while the interaction with the rest of the MPS sites is treated as an effective renormalized basis, whose size is referred to as the bond dimension. It is this parameter that determines the accuracy of a DMRG calculation and its computational cost.

While DMRG provides an efficient framework for obtaining stationary states in large Hilbert spaces, its extension to the time domain, the time-dependent density matrix renormalization group (TD-DMRG), allows for the real and imaginary propagation of quantum systems within the same tensor-network formalism. It enables the direct investigation of nonequilibrium and dynamical phenomena, as well as the calculation of time-dependent spectroscopic quantities such as autocorrelation functions and absorption cross sections. Importantly, TD-DMRG achieves this while efficiently handling large basis sets and complex entanglement structures. This allows for the time-dependent study of molecules with many correlated electrons and vibrational modes.^19–25^ Multiple time-dependent variants of the DMRG algorithm have been formulated over the years, such as time-evolving block decimation,^26,27^ adaptive TD-DMRG,^28–30^ the tensor-train split-operator Fourier transform,^31^ and the tangent-space formulation of TD-DMRG.^23^ The latter is especially suitable for DMRG variants employing MPSs as it exploits their compact structure. The tangent-space formulation of TD-DMRG achieves time evolution based on the Dirac–Frenkel variational principle,^32,33^ resulting in projecting the evolved wave function back onto the MPS manifold of a fixed maximum bond dimension.^34–36^ Since the entanglement entropy tends to increase with time, this procedure introduces truncation errors.^37,38^ Therefore, convergence of a TD-DMRG calculation with respect to the maximum bond dimension must be monitored. In this work, we apply the tangent-space TD-DMRG algorithm to the time evolution for a realistic *n*-mode quantized vibronic Hamiltonian. This idea has been previously explored by Shuai and co-workers while restricting the application of the *n*-mode quantization of anharmonic vibrational potentials to a single ground-state PES and treating off-diagonal terms as constants.^39,40^

In this work, we extend on this formalism by introducing two methodological innovations. We discuss the full *n*-mode quantization of all vibronic Hamiltonian terms, including the diagonal PESs and all off-diagonal nonadiabatic coupling terms. This generalizes our earlier vibronic DMRG work^23^ where the Hamiltonians employed relied on harmonic oscillator basis functions and a Taylor expansion in the canonical position and momentum operators. Moreover, we present a tailored MPS architecture with composite local operators removing the vacuum sector, thus reducing the dimensionality of the physical basis of the vibrational lattice sites and thereby enabling an efficient tensor-train representation of the vibronic wave function. The fully *n*-mode quantized Hamiltonian along with the MPS architecture introduced here constitute the first vibronic DMRG framework applicable to general anharmonic, multistate vibronic Hamiltonians, allowing for a wide variety of functional forms of the PESs and vibronic coupling terms. Finally, we demonstrate the applicability of these parametrized Hamiltonians in combination with TD-DMRG by obtaining accurate vibronic dynamics of a realistic molecular system.

## Theoretical Framework

### *n*-Mode Quantized Vibronic Hamiltonian

Vibronic dynamics involving multiple electronic states and vibrational degrees of freedom can be described with a general vibronic Hamiltonian of the following form

1 $$H_{vibronic}=\left[\begin{array}{cccc} H_{1}\left(Q\right) & V_{12}\left(Q\right) & \cdots & V_{1N_{el}}\left(Q\right)\\ V_{21}\left(Q\right) & H_{2}\left(Q\right) & & \\ \vdots & & \ddots & \\ V_{N_{el}1}\left(Q\right) & & & H_{N_{el}}\left(Q\right) \end{array}\right]$$

where $H_{\alpha }\left(Q\right)=T_{\alpha }\left(Q\right)+v_{\alpha }\left(Q\right)$ is the vibrational Hamiltonian associated with the αth electronic state (with a kinetic and a potential energy term, $T_{\alpha }\left(Q\right)$ and *v*_α_(***Q***), respectively). $V_{\alpha \beta }\left(Q\right)$ is the nonadiabatic coupling between the electronic states α and β. Indices α and β range from 1 to the number of electronic states *N*_el_. ***Q*** = {*Q*_1_, *Q*_2_, ..., *Q*_*M*_} denotes the set of *M* vibrational degrees of freedom of the system. Various approximations are made in such a model Hamiltonian, which enter through the specific definition of the vibrational Hamiltonians on the diagonal blocks in eq 1 and the nonadiabatic coupling terms on the off-diagonal blocks.

In a vibronic DMRG calculation, a second-quantized framework that allows for the implementation of arbitrary functional forms of the potential energy surfaces and nonadiabatic coupling terms is crucial. The *n*-mode quantization scheme offers a suitable approach for this purpose. The potential energy surfaces and the nonadiabatic coupling terms are expressed in a high-dimensional model representation, with the degrees of freedom corresponding to the vibrational modes of the system. The expansion is written as a sum over grouped terms, categorized by the number of degrees of freedom each term depends on.^11,13,41^ The *n*-mode expansion of an arbitrary function F(Q) depending on *M* degrees of freedom ***Q*** is given by

2 $$F\left(Q\right)=\sum_{i}^{M}F_{1}^{\left[i\right]}\left(Q_{i}\right)+\sum_{i<j}^{M}F_{2}^{\left[ij\right]}\left(Q_{i},Q_{j}\right)+\sum_{i<j<k}^{M}F_{3}^{\left[ijk\right]}\left(Q_{i},Q_{j},Q_{k}\right)+\cdot \cdot \cdot$$

$F_{1}^{\left[i\right]}\left(Q_{i}\right)$ depends on one internal coordinate and accounts for the variation of the function to be approximated with respect to that coordinate. The term $F_{2}^{\left[ij\right]}\left(Q_{i},Q_{j}\right)$ depends on two internal coordinates and accounts for the variation of the function with respect to a simultaneous change of the coordinates *Q*_*i*_ and *Q*_*j*_. Higher-order terms follow analogously. The vibrational Hamiltonian of an arbitrary electronic state and a nonadiabatic coupling term between two electronic states can be expressed in a second-quantized form in *n*-mode quantization as

3 $$H_{nmode}=\sum_{i=1}^{M}\sum_{k_{i},h_{i}=1}^{N_{i}}H_{k_{i},h_{i}}^{\left[i\right]}{\mathrm{b̂}}_{k_{i}}^{†}{\mathrm{b̂}}_{h_{i}}+\sum_{i=1}^{M}\sum_{i<j}^{M}\sum_{k_{i},h_{i}=1}^{N_{i}}\sum_{k_{j},h_{j}=1}^{N_{j}}H_{k_{i}k_{j},h_{i}h_{j}}^{\left[i,j\right]}{\mathrm{b̂}}_{k_{i}}^{†}{\mathrm{b̂}}_{k_{j}}^{†}{\mathrm{b̂}}_{h_{i}}{\mathrm{b̂}}_{h_{j}}+\cdot \cdot \cdot$$

and

4 $$V_{nmode}=\sum_{i=1}^{M}\sum_{k_{i},h_{i}=1}^{N_{i}}V_{k_{i},h_{i}}^{\left[i\right]}{\mathrm{b̂}}_{k_{i}}^{†}{\mathrm{b̂}}_{h_{i}}+\sum_{i=1}^{M}\sum_{i<j}^{M}\sum_{k_{i},h_{i}=1}^{N_{i}}\sum_{k_{j},h_{j}=1}^{N_{j}}V_{k_{i}k_{j},h_{i}h_{j}}^{\left[i,j\right]}{\mathrm{b̂}}_{k_{i}}^{†}{\mathrm{b̂}}_{k_{j}}^{†}{\mathrm{b̂}}_{h_{i}}{\mathrm{b̂}}_{h_{j}}+\cdot \cdot \cdot$$

respectively. Indices *i* and *j* run over the *M* vibrational modes and indices *k*_*i*_, *h*_*i*_, *k*_*j,*_ and *h*_*j*_ run over the number of single particle basis functions for vibrational modes *i* and *j*, respectively. The bosonic creation and annihilation operators are defined in ref 42. The one- and two-body integrals, in the case of a real-valued basis set, are defined as

5 $$H_{k_{i},h_{i}}^{\left[i\right]}=\int_{-\infty }^{+\infty }\phi_{i}^{k_{i}}\left(Q_{i}\right)\left[T\left(Q_{i}\right)+v_{1}^{\left[i\right]}\left(Q_{i}\right)\right]\phi_{i}^{h_{i}}\left(Q_{i}\right)dQ_{i}$$

6 $$H_{k_{i}k_{j},h_{i}h_{j}}^{\left[i,j\right]}=\int_{-\infty }^{+\infty }\int_{-\infty }^{+\infty }\phi_{i}^{k_{i}}\left(Q_{i}\right)\phi_{j}^{k_{j}}\left(Q_{j}\right)v_{2}^{\left[i,j\right]}\left(Q_{i},Q_{j}\right)\phi_{i}^{h_{i}}\left(Q_{i}\right)\phi_{j}^{h_{j}}\left(Q_{j}\right)dQ_{i}dQ_{j}$$

7 $$V_{k_{i},h_{i}}^{\left[i\right]}=\int_{-\infty }^{+\infty }\phi_{i}^{k_{i}}\left(Q_{i}\right)V_{1}^{\left[i\right]}\left(Q_{i}\right)\phi_{i}^{h_{i}}\left(Q_{i}\right)dQ_{i}$$

8 $$V_{k_{i}k_{j},h_{i}h_{j}}^{\left[i,j\right]}=\int_{-\infty }^{+\infty }\int_{-\infty }^{+\infty }\phi_{i}^{k_{i}}\left(Q_{i}\right)\phi_{j}^{k_{j}}\left(Q_{j}\right)V_{2}^{\left[i,j\right]}\left(Q_{i},Q_{j}\right)\phi_{i}^{h_{i}}\left(Q_{i}\right)\phi_{j}^{h_{j}}\left(Q_{j}\right)dQ_{i}dQ_{j}$$

where $T\left(Q_{i}\right)$ is the kinetic energy term of a vibrational mode *Q*_*i*_, *v*_1_^[*i*]^ and *v*_2_^[*i*,*j*]^ are the one- and two-body terms of the *n*-mode expansion of the potential energy surfaces, while $V_{1}^{\left[i\right]}\left(Q_{i}\right)$ and $V_{2}^{\left[i,j\right]}\left(Q_{i},Q_{j}\right)$ are the one- and two body terms of the *n*-mode expansion of the nonadiabatic coupling terms, respectively. Higher-order terms follow analogously. These integrals can be evaluated with an arbitrary set of single-particle basis functions $\left\{\phi_{i}^{k_{i}}\right\}$ , emphasizing the advantage of the second-quantized *n*-mode Hamiltonian, for which a suitable basis set can be chosen for a given problem. Since the *n*-mode expansion of a function rapidly converges for many systems with an appropriate choice of coordinates and functions,^43–45^ the *n*-mode quantized vibronic Hamiltonian in second quantization enables the efficient numerical description of high-dimensional vibronic problems. In particular, its compatibility with the second-quantized MPS/MPO formulation of DMRG makes it a powerful tool for exploring the time evolution and spectral properties of complex molecular systems.

### Vibronic Matrix Product State

The vibronic MPS was constructed as illustrated in Figure 1: the *N*_el_ electronic sites, which each correspond to an electronic state, are placed at the beginning of the DMRG lattice, followed by the *M* bosonic sites representing the vibrational modes of the system. The operators applied to the MPS sites are the electronic creation and annihilation operators ${â}_{\gamma }^{†}$ , ${â}_{\gamma }$ for the γth electronic state and the bosonic creation and annihilation operators ${\mathrm{b̂}}_{k_{i}}^{†}$ , ${\mathrm{b̂}}_{k_{i}}$ , which create and destroy occupations in the *k*_*i*_th vibrational basis function of the vibrational site corresponding to mode *i*. In a naive implementation, this would yield operators of dimension *d* = (*N*_*i*_ + 1) × (*N*_*i*_ + 1), where *N*_*i*_ is the number of vibrational basis functions describing a single vibrational mode *i*. Since an auxiliary vacuum state is needed to describe a depopulation from the basis function *k*_*i*_ to the vacuum or a population of a basis state *k*_*i*_ from the vacuum, the dimension of the matrices responsible for these operations has to be *N*_*i*_ + 1, rather than *N*_*i*_. By not registering the single bosonic operators separately, but the operator product of the form ${\mathrm{b̂}}_{k_{i}}^{†}{\mathrm{b̂}}_{h_{i}}$ as a composite operator (as it appears in eqs 3 and 4) renders the vacuum state redundant, resulting in product operators of dimension *d* = *N*_*i*_ × *N*_*i*_. The vibronic MPS is of U1 symmetry since there is electronic particle conservation.

**Figure 1:**
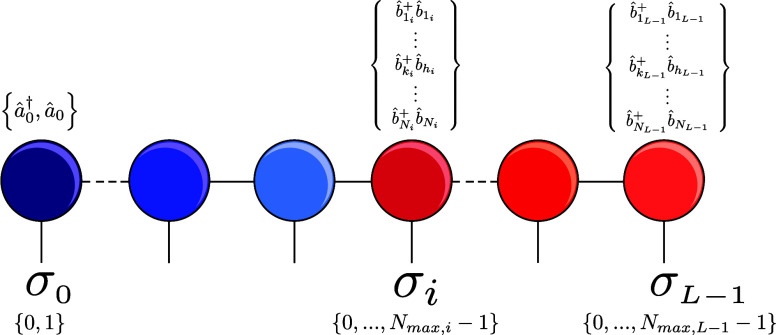
Graphical representation of the vibronic MPS. The first sites are electronic states (color-coded in blue) followed by the vibrational sites (color-coded in red). The electronic sites can have occupations 0 or 1 encoding whether the electronically excited state is populated or not. The quantum numbers of the physical basis σ of the vibrations can be any integer from 0 to *N*_max_ – 1, where *N*_max_ denotes the number of vibrational basis functions per mode. The corresponding operators acting on each site are indicated above the individual MPS sites.

This MPS differs significantly from the vibrational MPS we reported in previous work where a single MPS site corresponds to a single vibrational basis function.^42^ This is motivated by the fact that the description of molecules with a considerable number of vibrational modes and basis functions can result in long lattice sizes. In the Supporting Information to this article, we demonstrate that this vibronic MPS structure can lead to lower energies when employing the same bond dimension in time-independent energy optimizations.

### Measurements

The initial excited wavepacket |ψ_*e*_(0)⟩ is given by the transition dipole operator acting on the ground-state vibronic wave function ${\mathrm{\mu ̂}}_{ge}|\psi_{g}\left(0\right)\rangle$ . Assuming a coordinate independent transition dipole moment, the vibrational part of the initial wavepacket in the excited state is proportional to the vibrational wavepacket of the ground electronic state. This approximation is known as the Condon approximation and is valid for allowed electronic transitions and when the dipole moment varies only weakly upon a change in the nuclear coordinates.^46^ Both of these conditions are expected to hold for the *S*_0_ → *S*_4_ transition in maleimide. For an exact treatment of the dipole operator, one can also describe it in *n*-mode representation as

9 $$\mu_{ge}\left(Q\right)=\mu_{0}+\sum_{i}^{M}\mu_{ge,1}^{\left[i\right]}\left(Q_{i}\right)+\sum_{i<j}^{M}\mu_{ge,2}^{\left[ij\right]}\left(Q_{i},Q_{j}\right)+\sum_{i<j<k}^{M}\mu_{ge,3}^{\left[ijk\right]}\left(Q_{i},Q_{j},Q_{k}\right)+\cdot \cdot \cdot$$

The absorption spectra, assuming the dipole operator of eq 9 as constant, were obtained by Fourier transforming the autocorrelation function

10 $$I\left(\omega \right)\propto \int_{0}^{\infty }e^{i\omega t}C\left(t\right)\;dt=\int_{0}^{\infty }e^{i\omega t}\left\langle \psi \left(0\right)|\psi \left(t\right)\right\rangle \;dt$$

and subsequently plotting the absolute magnitude of the complex-valued quantity *I*(ω) after shifting the 0–0 transition to match the experimental excitation energy. Further postprocessing of the Fourier-transformed function, such as zero-padding or the convolution with a broadening function, was not necessary since this was not required to obtain results matching the experimental spectra. The population of the diabatic electronic states was measured by evaluating the expectation value of the electronic number operator

11 $${\mathrm{N̂}}_{\gamma }={â}_{\gamma }^{†}{â}_{\gamma }$$

where ${â}_{\gamma }^{†}$ and ${â}_{\gamma }$ are the creation and annihilation operators, respectively, acting on the electronic MPS site γ. Essentially, this projects the electronic part of the vibronic wave function onto the electronic state γ, yielding the probability of encountering the system in the diabatic electronic state γ.

### Computational Details

All quantum dynamics calculations were carried out with the DMRG software package QCMaquis.^47^ The time step for the time evolution should generally be chosen such that it captures the fastest relevant oscillations in the system under study. In our case, a time step of 0.5 fs delivered accurate results. A total of 800 sweeps (a sweep constitutes an optimization of each lattice site from the beginning of the lattice to its terminus and back) was employed in most calculations (unless stated otherwise), resulting in a total propagation time of 400 fs. For all calculations, the two-site DMRG integrator was employed. The integrals of eqs 5–8 that appear in the definition of the matrix product operator representation of the Hamiltonian were evaluated numerically. For these integrations, the basis functions were chosen to be the vibrational wave functions of the vibrational modes of the ground electronic state. This is a convenient choice since the initial wave function is easily representable in this basis. This choice may be less suited to systems in which the excited electronic states exhibit considerable Huang–Rhys factors. In such a case, it may be more advantageous to represent the wave function in a basis of displaced harmonic oscillators or displaced eigenfunctions of the vibrational Morse Hamiltonian. The initial wavepacket would then have to be transformed to this new basis.

## Results and Discussion

To demonstrate the application of the *n*-mode quantization framework for vibronic Hamiltonians with time-dependent tensor-network algorithms, we employ the tangent-space formulation of the time-dependent density matrix renormalization group method^23,35^ to calculate spectral properties of the maleimide molecule.^23,35,36^ The *S*_0_ → *S*_4_ transition was chosen for demonstration purposes of our framework, as it is the absorption band for which experimental spectra are available with good vibrational resolution, which is not the case for the first intense, but very broad *S*_0_ → *S*_3_ excitation band.^48^ The initial Franck–Condon excitation *S*_0_ → *S*_4_ serves as the starting point of the calculation, and the subsequent dynamics are described within the subspace of the *S*_3_ and *S*_4_ electronic states, which exhibit significant vibronic coupling. The *n*-mode quantized vibronic Hamiltonian employed for these two electronic excited states includes 6 of the total 24 vibrational modes. These six modes, along which cuts of the PES are depicted in Figure 2, were selected because they contribute the most to the nonadiabatic coupling terms between the selected electronic states. Hence, these are the essential vibrational modes necessary to reproduce the experimental spectrum. The vibrational modes are numbered in ascending order of magnitude of their respective harmonic frequencies. The parameters defining the vibronic Hamiltonian were taken from ref 49. As identified in that study, three of the included vibrational modes require anharmonic potentials to be described accurately, making maleimide a suitable system to evaluate our anharmonic formalism. The parametrized vibronic Hamiltonian includes up to two-body coupling terms; hence, the *n*-mode expansion of eq 2 and 3 only contains up to two-body expansion terms. Nonetheless, the formulation is general and higher-order terms of the expansion can be included in the vibronic DMRG calculation.

**Figure 2:**
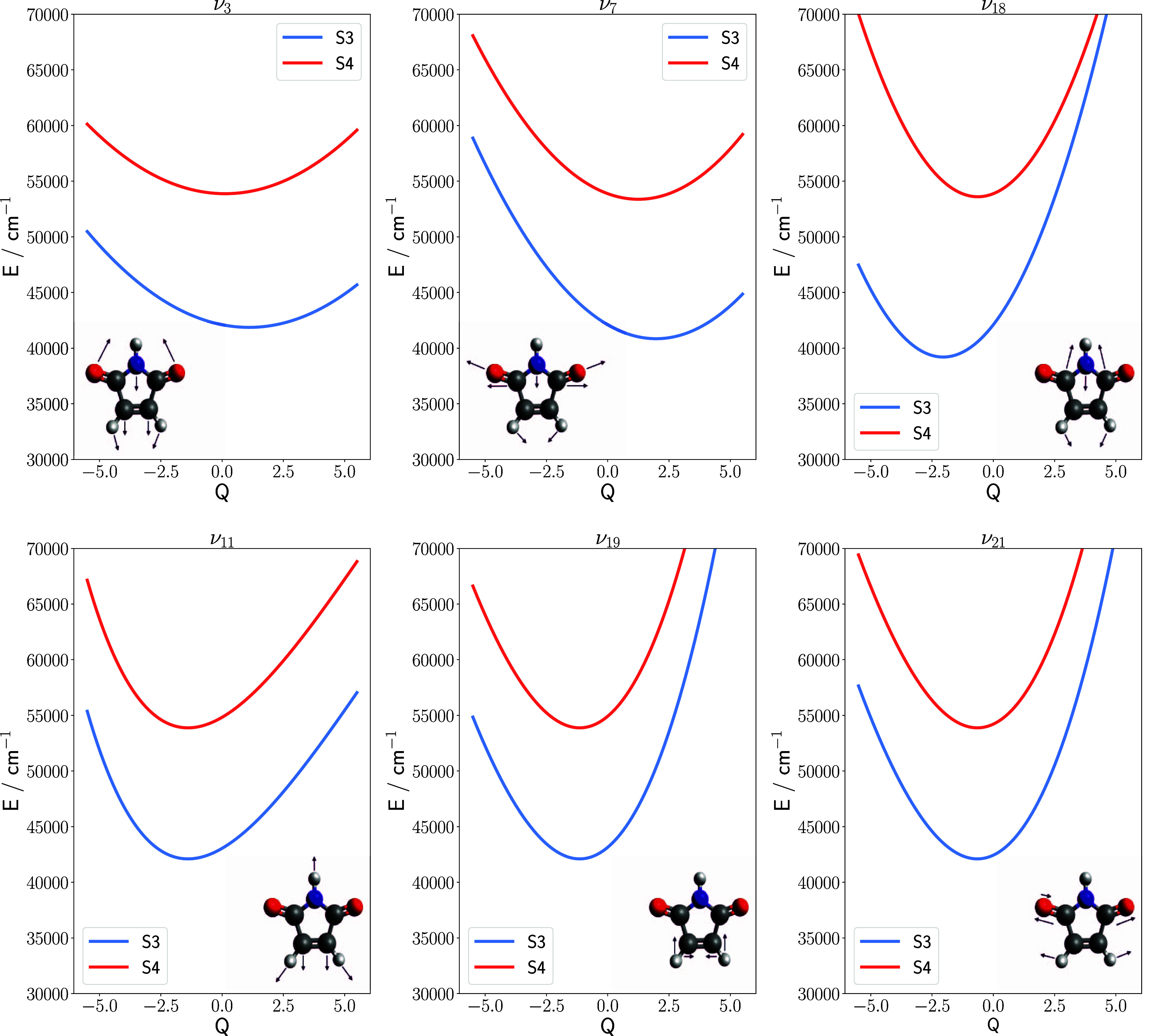
Cuts through the *S*_3_ and *S*_4_ potential energy surfaces along the selected vibrational mode coordinates. Vibrational mode indices are assigned according to the magnitude of the corresponding harmonic frequencies. Arrows attached to the molecular structures indicate the displacement of the atoms in each of the selected normal mode coordinate. Atom color code: gray—carbon, white—hydrogen, red—oxygen, blue—nitrogen.

To assess the reliability of our *n*-mode quantized vibronic framework, we calculated the absorption spectrum of maleimide by Fourier transforming the autocorrelation function obtained by a TD-DMRG calculation of a total propagation time of 800 fs with a maximum bond dimension of 75 according to eq 10, shown in Figure 3. The calculated spectrum is in good agreement with the experimental results taken from ref 49. Accordingly, this agreement suggests that the essential vibronic couplings and anharmonicities are well described within our framework, allowing for reliable predictions of the spectral line shapes and peak positions. We also note that our results are consistent with state-of-the-art multilayer multiconfigurational time-dependent Hartree (ML-MCTDH) calculations performed in ref 49.

**Figure 3:**
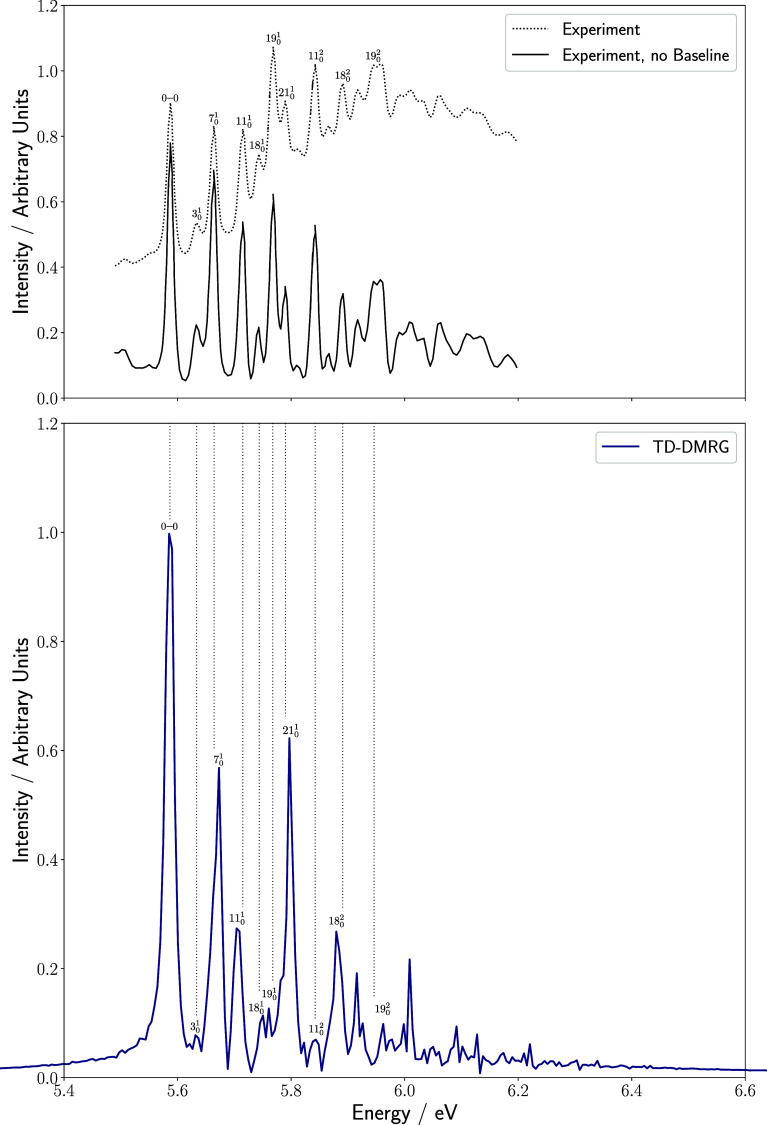
Comparison of the experimental gas-phase absorption spectrum of maleimide taken from ref 49 (top) and the TD-DMRG spectrum obtained in this work (with a time step of 0.5 fs, a total propagation time of 800 fs, and a bond dimension of 75). Top: two experimental curves are shown. One represents the raw data and the other shows the spectrum with its envelope subtracted to allow for a better comparison with the calculated spectrum. All fundamental transitions as well as some overtones are labeled according to ref 49. Dotted vertical lines connect the experimental values of these transitions with the calculated results.

Since the key convergence parameter of a DMRG calculation is the bond dimension, autocorrelation functions were obtained for different values thereof, as shown in Figure 4. Although the autocorrelation functions initially coincide, they diverge at longer propagation times. This behavior arises from the fact that the variation of the wave function |ψ­(*t*)⟩ after a time step Δ*t* is proportional to H|ψ(t)⟩×Δt , if Δ*t* ≠ 0. In that case, the exact tensor-train representation of H|ψ(t)⟩ requires a bond dimension equal to the product of the MPO and MPS bond dimensions. As a result, an accurate and long-time propagation demands increasingly larger MPS bond dimensions if the wave function exhibits considerable entanglement between distant MPS lattice sites. The time evolution of the bond dimension growth and its subsequent truncation by projecting the evolved MPS back onto the manifold spanned by all MPSs of a given bond dimension are illustrated in Figure 5.

**Figure 4:**
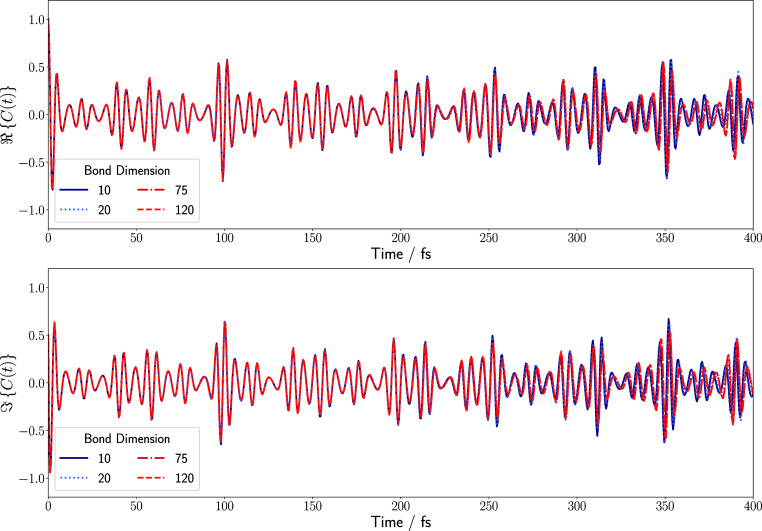
Real and imaginary parts of the autocorrelation function obtained by a TD-DMRG calculation of maleimide upon photoexcitation onto the *S*_4_ surface with different values for the maximum bond dimension. The top panel shows the real and the bottom panel shows the imaginary part of the autocorrelation function. The results were obtained by employing a time step of 0.5 fs and propagating the wave function for 400 fs.

**Figure 5:**
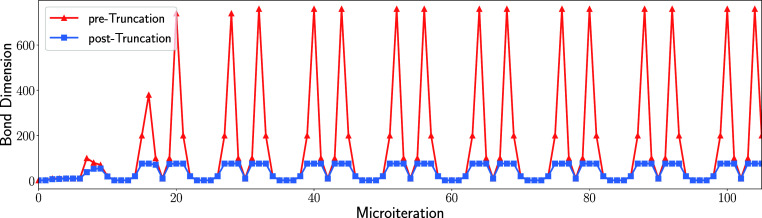
Non-truncated and truncated bond dimensions of the MPS over the first 100 microiterations of the TD-DMRG calculation. The data were obtained by a calculation employing a 0.5 fs time step and a maximum bond dimension of 75.

For the maleimide system studied in this work, comprised of two coupled electronic states and six vibrational degrees of freedom, a maximum bond dimension of 75 suffices to capture all relevant quantum dynamics. The autocorrelation function has converged for this calculation, exemplified by the near-perfect agreement of the autocorrelation function obtained by a calculation with maximum bond dimensions of 75 and 120. For shorter time propagations, smaller bond dimensions are sufficient. For propagations up to 200 fs, converged calculations can be obtained with a modest bond dimension of 10, exemplified by the fact that the resulting autocorrelation functions obtained by TD-DMRG calculations with larger bond dimensions match the one obtained with a bond dimension of 10. Additionally, in the absorption spectrum obtained by the Fourier transform of the autocorrelation function calculated with a maximum bond dimension of 75, all fundamental transitions along with the most prominent overtones are present, as illustrated in Figure 6. Performing calculations with bond dimensions significantly lower than 75, the 3_0_^1^ transition is lacking in the calculated absorption spectra. Among the vibrational modes of maleimide, ν_3_ exhibits the smallest linear displacement between the *S*_0_ and *S*_4_ PESs. Since the ground-state vibrational wave function of the *S*_0_ electronic state coincides largely with the node of the first vibrationally excited state of the *S*_4_ PES, the resulting transition intensity is low in the absence of interstate coupling. Consequently, this results in an inherently weak transition intensity governed by the Franck–Condon overlap between the initial and final states of a given transition. Moreover, this vibrational mode mediates electronic coupling between *S*_3_ and *S*_4_ the strongest, giving rise to pronounced entanglement between the electronic MPS sites and the one corresponding to ν_3_. Accurately capturing this correlated character requires a sufficiently large bond dimension within the tensor-network representation. At low bond dimensions, the MPS representation remains in a mean-field-like state where these degrees of freedom are largely decoupled and therefore fails to recover the correlation-induced intensity of the transition.

**Figure 6:**
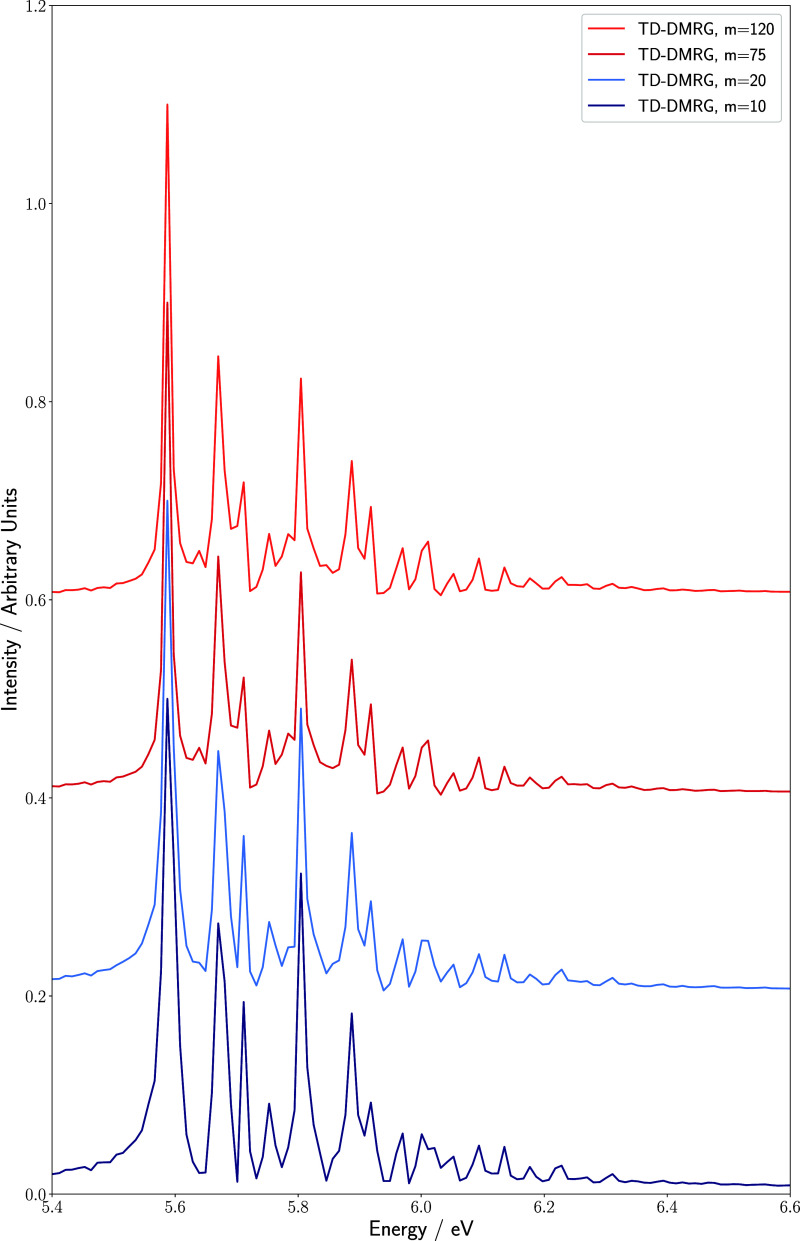
Absorption spectrum of the maleimide molecule upon a Franck–Condon excitation to the *S*_4_ electronic surface for different values of the maximum bond dimension m. All calculations were conducted with a time step of 0.5 fs for a total propagation time of 400 fs.

The diabatic state populations of the *S*_3_ and *S*_4_ electronic states have been monitored throughout the time propagation by evaluating the expectation value of the electronic number operator of eq 11. They are shown in Figure 7. The wavepacket is initialized on the *S*_4_ potential energy surface at *t* = 0. Throughout the propagation, a small fraction of the initial population of the *S*_4_ state is lost to the *S*_3_ state due to nonzero terms in the off-diagonal block in the vibronic Hamiltonian. These terms do not exhibit large magnitudes, explaining the slow population transfer. The population dynamics are consistent with reference calculations obtained with the ML-MCTDH method.^49^

**Figure 7:**
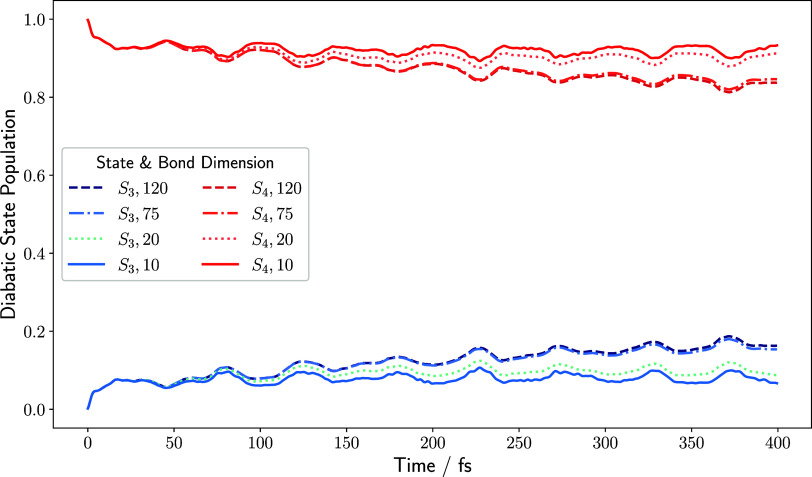
Evolution of the diabatic state population of the maleimide molecule upon photoexcitation onto the *S*_4_ electronic state for various values of the maximum bond dimension. The results were obtained for a time step of 0.5 fs for a total propagation time of 400 fs.

Increasing the bond dimension is not the only route to improve the accuracy. Since the integrals corresponding to the diagonal one- and two-body terms of the vibrational Hamiltonians $H_{k_{i},h_{i}}$ and $H_{k_{i}k_{j},h_{i}h_{j}}$ , as well as the off-diagonal nonadiabatic coupling terms $V_{k_{i},h_{i}}$ and $V_{k_{i}k_{j},h_{i}h_{j}}$ , defined in eqs 3 and 4, are evaluated numerically, finer grid spacing and larger integration bounds could also enhance accuracy. Moreover, increasing the local Hilbert space dimension *N*_max_ of each vibrational MPS site reduces the extent of finite basis size errors by including more vibrational basis functions. This effect is illustrated in Figure 8, which shows autocorrelation functions and absorption spectra calculated with the same bond dimension but different values of *N*_max_. Similar to the effect a larger maximum bond dimension has on the quality of the calculated time-dependent quantities, a larger local Hilbert space also enhances the accuracy of a TD-DMRG calculation. The resulting autocorrelation functions initially coincide but diverge at longer propagation times for different values of *N*_max_. The calculated absorption spectra become more accurate with increasingly large physical basis sizes. This is emphasized by the appearance of the signal corresponding to the 3_0_^1^ transition in the calculations with larger local Hilbert spaces. The shape of the *S*_3_ PES along the ν_3_ coordinate poses considerable computational demand due to its large displacement with respect to the ground-state minimum energy point, as well as exhibiting the largest frequency correction with respect to the ground state of all the vibrational modes of the *S*_4_ PES. Capturing relevant regions of this strongly shifted surface requires a large local basis set. Moreover, extending the local Hilbert space facilitates the inclusion of higher *S*_3_ vibrational excitations that become partially resonant with low-lying *S*_4_ vibronic levels, allowing proper mixing and hence a balanced description of the transition energy and intensity.

**Figure 8:**
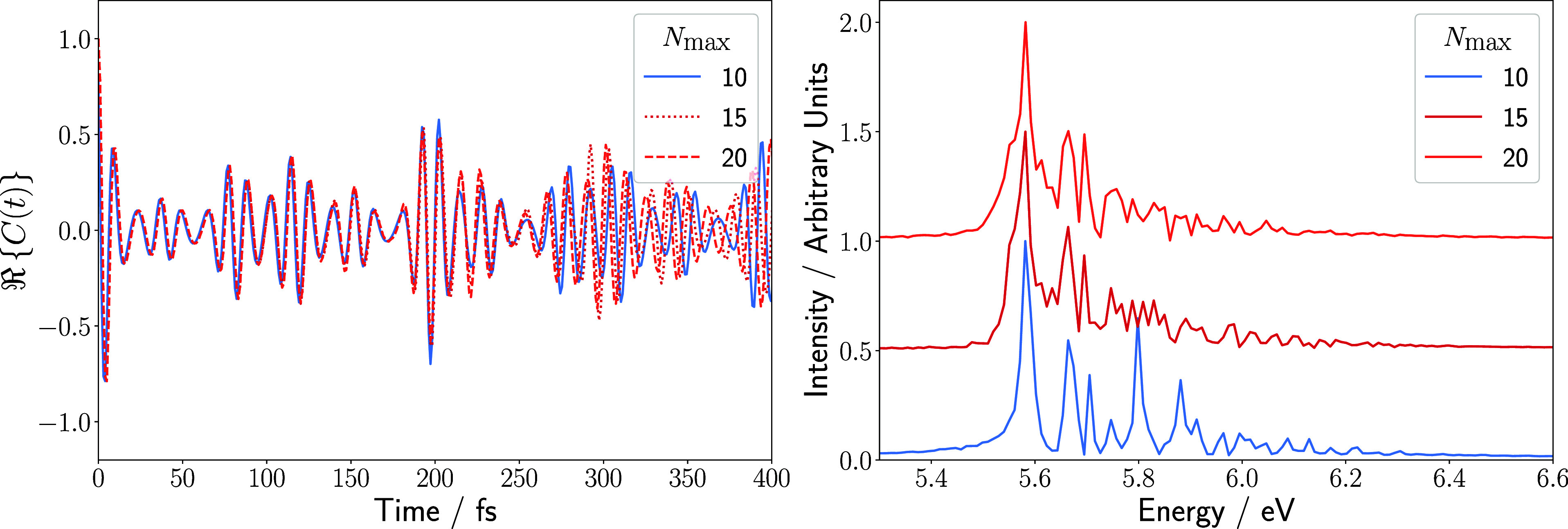
Real part of the autocorrelation function (left) and absorption spectra (right) obtained by a TD-DMRG calculation with a bond dimension of 10 and a time step of 0.5 fs for a total propagation time of 400 fs for different local Hilbert space sizes *N*_max_.

Although increasing *N*_max_ and the chosen maximum bond dimension improve the accuracy, these parameters should be chosen judiciously. In contrast to ground-state DMRG optimizations, which often converge within a few iterations, time-dependent calculations involve many time steps, each corresponding to a sweep, which constitutes the optimization of each MPS lattice site from left to right and back. Consequently, the total computational cost scales with both the bond dimension and the local basis size. It is therefore important to strike a balance between accuracy and computational feasibility by selecting parameters that yield efficient calculations while still capturing all relevant spectral features.

In this work, our framework was applied to the maleimide system to examine its reliability and convergence behavior under well-controlled conditions. However, the methodology is general and can be readily extended to more complex molecular systems. For larger systems with more electronic states and vibrational modes, or in cases with stronger vibronic coupling and MPSs showing larger intersite entanglement, larger bond dimensions will be necessary to accurately represent the increased complexity of the wave function.

## Conclusions

In this work, we applied the *n*-mode quantization framework to a vibronic Hamiltonian of a molecule whose photochemical properties are governed by multiple anharmonic potential energy surfaces. In principle, this framework also allows nonadiabatic coupling terms to include complex functional forms. The *n*-mode quantized vibronic Hamiltonian employed with the TD-DMRG algorithm provides an accurate description of vibronic dynamics with significant correlations in the vibrational and vibronic parts of the wave function that could not be captured efficiently by harmonic approaches.

Conventional vibronic DMRG methods based on canonical quantization employ harmonic oscillator basis functions and represent the PES through a Taylor expansion around a reference molecular structure. Accurately describing strongly anharmonic systems within this canonical harmonic-oscillator-based framework requires a very large number of single-particle basis functions since the harmonic expansion poorly approximates regions far from the reference point. Consequently, the solution of the Schrödinger equation can become computationally intractable even for moderately sized molecules that exhibit pronounced anharmonicities. Moreover, since a Taylor expansion is intrinsically local, complex potential energy surfaces, such as double-well or multi-minima surfaces, cannot be reliably represented.

Expressing the vibronic wave function as a tensor network allows us to mitigate the curse of dimensionality that limits quantum mechanical many-body methods. Applying our approach to the photoinduced dynamics of maleimide allowed us to study how the bond dimension *m* and the number of single-particle basis functions per mode *N*_max_, which both govern the accuracy and cost of a vibronic DMRG calculation, should be chosen to achieve convergence for moderately sized molecules. Other state-of-the-art methods for vibronic dynamics include ML-MCTDH^50–54^ and surface-hopping approaches.^55–57^ In contrast to surface hopping, our method offers a fully quantum-mechanical description of the coupled electronic and nuclear degrees of freedom. Compared to ML-MCTDH, the *n*-mode quantized vibronic Hamiltonian in combination with TD-DMRG provides a more rigorous and systematic error control through tuning of the bond dimension and monitoring of the discarded singular values that arise during the truncation steps intrinsic to DMRG.

The combination of a general *n*-mode quantized vibronic Hamiltonian with MPS representations constitutes the main conceptual advance of this work. This framework removes previous limitations, such as harmonic approximations, local truncated PES expansions, and simplified interstate couplings. It enables a systematically improvable description of vibronic dynamics within DMRG.

While a systematic scaling analysis was not performed, a thorough analysis of how the framework described in this work would compare to MCTDH and the vibronic DMRG approach based on canonical quantization would be beneficial. However, evidence that this approach would scale decently with an increased number of vibrational modes and a higher dimensional *n*-mode expansion is given in ref 42 where vibrationally excited states of methyloxirane were calculated with an *n*-mode quantized PES containing up to three-body terms along with 24 vibrational modes. In *n*-mode quantized vibronic DMRG, the lattice size would only be increased by the number of excited electronic states, compared to the purely vibrational case, whose corresponding lattice site would have a manageable physical dimension of 2.

While tensor-network methods have opened the door to treating larger anharmonic vibrational and vibronic systems, increasing system size remains a significant challenge. Further improvements can be achieved by applying concepts from quantum information theory to optimize the mapping and ordering of vibrational modes and electronic basis functions on the DMRG lattice. Such analyses have already been demonstrated to be very useful for purely electronic^58–60^ and purely vibrational Hamiltonians^61^ and will serve as a foundation for extending the accessible system size in vibronic calculations. In addition, finite-temperature DMRG algorithms can be incorporated with minimal modifications to the present framework,^62,63^ enabling the simulation of temperature-dependent spectroscopic features relevant to actual experimental conditions.

## Supplementary Material



## Data Availability

All source data of the figures, input and output files generated for this work, including autocorrelation functions and population dynamics, will be made available on Zenodo upon publication of this work.

## Notes

Input file and integrals for the QCMaquis DMRG calculations (PDF)
