# Supplementary material for: n‑Mode Quantized Anharmonic Vibronic Hamiltonians for Matrix Product State Dynamics
Source: J Chem Theory Comput. 2026 Feb 12;22(4):1896–906. doi: 10.1021/acs.jctc.5c02014 (PMC12937108; doi:10.1021/acs.jctc.5c02014)
Supplement: Supplementary file 1 [file ct5c02014_si_001.pdf]

Supplementary Information for Article : N-Mode Quantized Anharmonic Vibronic Hamiltonians for Matrix Product State Dynamics

Valentin Barandun,[†] Nina Glaser,[‡] and Markus Reiher[*,†]

†*ETH Zürich, Department of Chemistry and Applied Biosciences, Vladimir-Prelog-Weg 2, 8093 Zürich, Switzerland*

‡*NNF Quantum Computing Programme, Niels Bohr Institute, University of Copenhagen, Blegdamsvej 21, 2100 København Ø, Denmark*

E-mail: mreiher@ethz.ch

Raw Data & Code Availability

All data that was generated for this work, including autocorrelation functions and population dynamics, along with the code used to generate all data presented in this study is available under the following Zenodo DOI: 10.5281/zenodo.17233386.

Variational Analysis of the MPS structure

We provide a comparison between the MPS architecture in this work and the MPS structure previously reported in Ref. 40. The data labeled as 'paired' in Fig.1 represents the results with the MPS employed in this study (omitting the electronic degrees of freedom). Fig.1 demonstrates the converged ground state energies of a vibrational DMRG optimization for different values of the maximum bond dimensions. The studied system is the n-mode

vibrational Hamiltonian of ethene, consisting of 12 vibrational modes described by 6 basis functions each. The results demonstrate that the compact MPS structure applied here yields variationally improved energies compared to the 'extended N-mode' MPS described in Ref. 40.

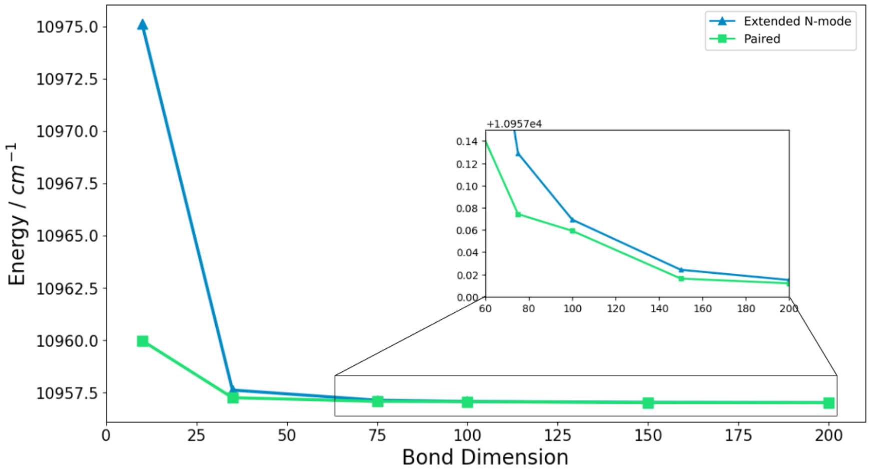

Figure 1: Comparison of the variational ground state vibrational energy for different values of the maximum bond dimensions obtained with two different MPS architectures. The data in blue was obtained by a vDMRG calculation with the vibrational MPS reported in Ref. 40, where the data in bright teal corresponds to results from vDMRG calculations employing the MPS architecture used in this work.

QCMaquis Input File

The following is a QCMaquis input file used for the calculations presented in this publication. In order to generate all data shown in this manuscript, only the maximum bond dimension and size of the local Hilbert space was varied from calculation to calculation. This can be achieved by changing the parameters `max_bond_dimension` and `Nmax`.

```
// -- Generic DMRG parameters --
simulation_type                = "evolve"
nsweeps                        = 800
ngrowsweeps                    = 4
nmainsweeps                    = 5
max_bond_dimension             = 20
optimization                   = twosite
truncation_initial             = 1.0E-16
```

```
truncation_main               = 1.0E-18
truncation_final              = 1.0E-20
alpha_initial                 = 1.0E-12
alpha_main                    = 0.
alpha_final                   = 0.
resultfile                    = 'res_maleimide.h5'
//chkpfile                    = 'chkp_maleimide.h5'
L                             = 8
symmetry                      = 'u1'
COMPLEX                       = 1

// -- Definition of the initial state for the propagation --
init_type                     = 'basis_state_generic'
init_basis_state              = '0,1,0,0,0,0,0,0'

// -- Vibronic Hamiltonian defininition --
LATTICE                       = "vibronic lattice"
MODEL                         = vibronicgeneric
model_library                 = coded
vibronic_num_elestates        = 2
vibronic_num_vibmodes         = 6
Nmax                          = 10
integral_file                 = FCIDUMP_maleimide_ene

// -- Parameters for the propagation --
propagator_maxiter            = 30
propagator_accuracy           = 1.0E-10
time_step                     = 0.5
time_units                    = "fs"
hamiltonian_units             = "cm-1"
imaginary_time                = "no"
TD_backpropagation            = "yes"
TD_noise                      = "yes"

// -- Measurements --
MEASURE[Population]           = 1
MEASURE[Autocorrelation]      = 1
measure_each                  = 1
ALWAYS_MEASURE                = "PopulationState0,PopulationState1,
                                 Autocorrelation"
```

Maleimide Integral File

The file is organized into sections separated by headers of the form `EL_ST X Y`, where `X` and `Y` are integers smaller than N_{el}. When the parser encounters a header of this type, all parameters listed until the next header are interpreted as belonging either to the vibrational Hamiltonian of electronic state `X` (if $\mathtt{X} = \mathtt{Y}$) or to the nonadiabatic coupling between electronic states `X` and `Y` (if $\mathtt{X} \neq \mathtt{Y}$). For example, the header `EL_ST 0 0` indicates that the lines listed below correspond to the 0–0 block of the Hamiltonian, i.e. in the case of our studied system, to the vibrational Hamiltonian of the S_3 state of maleimide. Note that the definition of the vibrational Hamiltonian for a given pair of `X` and `Y` values does not need to appear in a single continuous block; it can instead be split across multiple sections of the file. In the case a block describes an on-diagonal term, the energy offset of this electornic state can be defined by its value followed by two zeros, eg. E_{off} 0 0. After the header, the definition of the respective Hamiltonian blocks follow. Each line corresponds to a term in the n-mode quantized vibrational Hamiltonian of a given electronic state, or in the case of an off-diagonal block, to an n-mode quantized nonadiabatic coupling term. In this integral file format, the indices that contain dashes correspond to the mode (first number) and modal (second number) indicices of the creation and annihilation operators in the n-mode quantized Hamiltonian. For example, an entry like `1-0 1-0` corresponds to a term $\hat{b}^{\dagger}_{0_1}\hat{b}_{0_1}$. Similarly the two-body terms are indicated by 4 terms with dashes. For example `1-0 1-0 2-0 2-0` corresponds to $\hat{b}^{\dagger}_{0_1}\hat{b}^{\dagger}_{0_2}\hat{b}_{0_1}\hat{b}_{0_2}$. The last entry in each line is the value of the one- and two-body integrals $H^{[i]}_{k_i,h_i}, H^{[i,j]}_{k_ik_j,h_ih_j}$ or $\mathcal{V}^{[i]}_{k_i,h_i}, \mathcal{V}^{[i,j]}_{k_ik_j,h_ih_j}$.

```
EL_ST 0 0
0.0 0 0
197.61038653234073 1-0 1-0
307.99912196735653 1-0 1-1
0.03479535367853259 1-0 1-2
-9.43163597128945e-05 1-0 1-3
8.07002899870644e-05 1-0 1-4
2.3714791318579786e-09 1-0 1-5
```

2.246944682315656e-09 1-0 1-6

-1.8776293634192866e-09 1-0 1-7

-1.4392166955162078e-09 1-0 1-8

1.5800970482648957e-09 1-0 1-9

307.99912196738177 1-1 1-0

592.8311595632085 1-1 1-1

435.5764265697192 1-1 1-2

-0.0601059451589947 1-1 1-3

0.00018863672481384523 1-1 1-4

0.0001804565235107486 1-1 1-5

1.0873060426536132e-09 1-1 1-6

1.5171015787459893e-09 1-1 1-7

-4.658224552758739e-10 1-1 1-8

9.518993812626343e-10 1-1 1-9

0.03479535372792952 1-2 1-0

435.5764265697283 1-2 1-1

988.0519325658965 1-2 1-2

-533.4698611594195 1-2 1-3

0.08477444068574869 1-2 1-4

0.0002982609550969164 1-2 1-5

0.0003125595100900951 1-2 1-6

2.138354758304824e-09 1-2 1-7

6.673386803071917e-09 1-2 1-8

-4.629815942008754e-09 1-2 1-9

-9.431642637309988e-05 1-3 1-0

-0.06010594506736311 1-3 1-1

-533.4698611595961 1-3 1-2

1383.2727055042722 1-3 1-3

-615.9977818704818 1-3 1-4

-0.10914871737696785 1-3 1-5

-0.0004218048642446444 1-3 1-6

-0.0004774513033204399 1-3 1-7

3.841113534496627e-09 1-3 1-8

-5.495885823379148e-09 1-3 1-9

8.07001798287817e-05 1-4 1-0

0.00018863673159659835 1-4 1-1

0.0847744406390234 1-4 1-2

-615.997781870426 1-4 1-3

1778.4934784094517 1-4 1-4

688.7062849389675 1-4 1-5

0.1333185312224714 1-4 1-6

0.0005579890054719924 1-4 1-7

0.0006752165661945384 1-4 1-8

-3.53183557799318e-09 1-4 1-9

2.251747132560669e-09 1-5 1-0

0.00018045649225610673 1-5 1-1

0.00029826108359906313 1-5 1-2

-0.10914871736326859 1-5 1-3

688.7062849390226 1-5 1-4

2173.7142512117834 1-5 1-5

754.4397468725211 1-5 1-6

0.1573176861123784 1-5 1-7

0.0007058066743204743 1-5 1-8

0.0009058943668858535 1-5 1-9

2.213763822072898e-09 1-6 1-0

9.480233537413621e-10 1-6 1-1

0.0003125594336543424 1-6 1-2

-0.0004218048404251993 1-6 1-3

0.13331853126362603 1-6 1-4

754.4397468726637 1-6 1-5

2568.93502400513 1-6 1-6

814.8878582535402 1-6 1-7

0.1811618492776006 1-6 1-8

0.000864422225440159 1-6 1-9

-1.8104179366452284e-09 1-7 1-0

1.5688674275631926e-09 1-7 1-1

1.9562075013562845e-09 1-7 1-2

-0.0004774511982253269 1-7 1-3

0.0005579890091797103 1-7 1-4

0.15731768619218656 1-7 1-5

814.8878582536465 1-7 1-6

2964.155796721331 1-7 1-7

871.1515462545522 1-7 1-8

0.20485925706839225 1-7 1-9

-1.3799609722931503e-09 1-8 1-0

-3.45011978148572e-10 1-8 1-1

6.62376528485418e-09 1-8 1-2

3.7881891753103806e-09 1-8 1-3

0.0006752165410209723 1-8 1-4

0.0007058066289283523 1-8 1-5

0.1811618492257594 1-8 1-6

871.151546254614 1-8 1-7

3359.3765694160247 1-8 1-8

923.9955176551272 1-8 1-9

1.635151593760432e-09 1-9 1-0

1.0164793630721255e-09 1-9 1-1

-4.613926949516341e-09 1-9 1-2

-5.552926385428433e-09 1-9 1-3

-3.515200365693293e-09 1-9 1-4

0.000905894394102161 1-9 1-5

0.0008644222252491891 1-9 1-6

0.2048592570912433 1-9 1-7

923.9955176549322 1-9 1-8

3754.5973420590312 1-9 1-9

322.6292025025035 2-0 2-0

901.1826161264164 2-0 2-1

0.05680874291928717 2-0 2-2

-0.0002759651676173804 2-0 2-3

0.00013175482356390505 2-0 2-4

3.588772285079144e-09 2-0 2-5

3.965263576535816e-09 2-0 2-6

-2.92170268982872e-09 2-0 2-7

-2.516725720752567e-09 2-0 2-8

2.4674379601741264e-09 2-0 2-9

901.1826161264577 2-1 2-0

967.8876074414457 2-1 2-1

1274.4643592262303 2-1 2-2

-0.09813215168657052 2-1 2-3

0.0005519346718721709 2-1 2-4

0.0002946229120358357 2-1 2-5

1.8411355301228844e-09 2-1 2-6

2.4121664320301808e-09 2-1 2-7

-9.144839030250555e-10 2-1 2-8

1.7492418393585595e-09 2-1 2-9

0.05680874299994798 2-2 2-0

1274.464359226245 2-2 2-1

1613.1460123620034 2-2 2-2

-1560.8932974659854 2-2 2-3

0.1384072476818119 2-2 2-4

0.0008726859769002661 2-2 2-5

0.0005103004930819542 2-2 2-6

2.7939959978962237e-09 2-2 2-7

1.1723437284755151e-08 2-2 2-8

-7.220211096114779e-09 2-2 2-9

-0.00027596527647654286 2-3 2-0

-0.0981321515369018 2-3 2-1

-1560.893297466274 2-3 2-2

2258.404417158723 2-3 2-3

-1802.3638802843186 2-3 2-4

-0.17820197910259594 2-3 2-5

-0.00123416058973338 2-3 2-6

-0.0007795144717961401 2-3 2-7

5.981515793384656e-09 2-3 2-8

-8.79831135702005e-09 2-3 2-9

0.00013175464369363636 2-4 2-0

0.0005519346829118315 2-4 2-1

0.13840724760552803 2-4 2-2

-1802.3638802842274 2-4 2-3

2903.6628219239738 2-4 2-4

2015.1035744391077 2-4 2-5

0.21766291512244607 2-4 2-6

0.0016326327221699685 2-4 2-7

0.0011023951342586675 2-4 2-8

-4.454780560028201e-09 2-4 2-9

3.3932952582527148e-09 2-5 2-0

0.00029462286096521646 2-5 2-1

0.0008726861867816193 2-5 2-2

-0.17820197908031332 2-5 2-3

2015.1035744391975 2-5 2-4

3548.92122645342 2-5 2-5

2207.434814916558 2-5 2-6

0.2568452052350949 2-5 2-7

0.002065138890240896 2-5 2-8

0.0014790129012918884 2-5 2-9

3.9110878433119835e-09 2-6 2-0

1.613732085881115e-09 2-6 2-1

0.0005103003683059994 2-6 2-2

-0.0012341605508549305 2-6 2-3

0.21766291518974867 2-6 2-4

2207.434814916791 2-6 2-5

4194.179631022125 2-6 2-6

2384.30151118158 2-6 2-7

0.29577445874133446 2-6 2-8

0.0025292430724714785 2-6 2-9

-2.8119773248624908e-09 2-7 2-0

2.4966850037429034e-09 2-7 2-1

2.496547612329556e-09 2-7 2-2

-0.0007795143001275971 2-7 2-3

0.001632632728194964 2-7 2-4

0.25684520536515265 2-7 2-5

2384.3015111817535 2-7 2-6

4839.438035431233 2-7 2-7

2548.924894599486 2-7 2-8

0.33446409873022276 2-7 2-9

-2.4199752299492605e-09 2-8 2-0

-7.172591287268786e-10 2-8 2-1

1.1642455188096713e-08 2-8 2-2

5.895120277043613e-09 2-8 2-3

0.0011023950932182761 2-8 2-4

0.0020651388161313095 2-8 2-5

0.29577445865675145 2-8 2-6

2548.924894599587 2-8 2-7

5484.69643984363 2-8 2-8

2703.542440553079 2-8 2-9

2.5573347143703653e-09 2-9 2-0

1.8546679587169715e-09 2-9 2-1

-7.194272711552338e-09 2-9 2-2

-8.891389502298442e-09 2-9 2-3

-4.427629730265967e-09 2-9 2-4

0.0014790129456318404 2-9 2-5

0.002529243072099077 2-9 2-6

0.33446409876751204 2-9 2-7

2703.5424405527606 2-9 2-8

6129.954844171233 2-9 2-9

693.6527853732235 3-0 3-0

-2007.6979802293824 3-0 3-1

0.12213877529165984 3-0 3-2

0.0006148222111847907 3-0 3-3

0.00028328031814253956 3-0 3-4

1.0519404032398593e-08 3-0 3-5

5.585820043459282e-09 3-0 3-6

-7.705917695539632e-09 3-0 3-7

-3.7571773223890615e-09 3-0 3-8

6.417294741756222e-09 3-0 3-9

-2007.6979802292938 3-1 3-0

2080.9583560851847 3-1 3-1

-2839.313002853668 3-1 3-2

-0.21098416252573315 3-1 3-3

-0.0012296132805015988 3-1 3-4

0.0006334390977954098 3-1 3-5

3.30567821291927e-09 3-1 3-6

5.826954311407671e-09 3-1 3-7

-4.412965403177912e-10 3-1 3-8

1.8282775573962496e-09 3-1 3-9

0.12213877546508911 3-2 3-0

-2839.313002853636 3-2 3-1

3468.263926484021 3-2 3-2

3477.4331690355793 3-2 3-3

0.29757560645771264 3-2 3-4

-0.001944192540168307 3-2 3-5

0.0010971534675381298 3-2 3-6

1.291261883423762e-08 3-2 3-7

1.700317934057295e-08 3-2 3-8

-1.8877696251596312e-08 3-2 3-9

0.0006148219772063365 3-3 3-0

-0.2109841622041131 3-3 3-1

3477.433169034959 3-3 3-2

4855.569496803194 3-3 3-3

4015.392948462796 3-3 3-4

-0.3831343387819288 3-3 3-5

0.002749471489901058 3-3 3-6

-0.0016759349206553558 3-3 3-7

1.5728843348025093e-08 3-3 3-8

-2.0645370182376103e-08 3-3 3-9

0.0002832799314378044 3-4 3-0

-0.0012296132567052218 3-4 3-1

0.2975756062937762 3-4 3-2

4015.3929484629916 3-4 3-3

6242.875066829586 3-4 3-4

-4489.344672102118 3-4 3-5

0.4679751981395839 3-4 3-6

-0.0036372478982216786 3-4 3-7

0.0023701422045768355 3-4 3-8

-2.25676051071085e-08 3-4 3-9

1.0099112304283452e-08 3-5 3-0

0.0006334389880283948 3-5 3-1

-0.0019441920889713591 3-5 3-2

-0.383134338733953 3-5 3-3

-4489.344672101925 3-5 3-4

7630.180637021414 3-5 3-5

-4917.829461042648 3-5 3-6

0.5522171573902597 3-5 3-7

-0.004600820415952439 3-5 3-8

0.003179860998734342 3-5 3-9

5.46937774416483e-09 3-6 3-0

2.8167142228413522e-09 3-6 3-1

0.001097153199111376 3-6 3-2

0.0027494715737256246 3-6 3-3

0.4679751982846483 3-6 3-4

-4917.829461042148 3-6 3-5

9017.486206763504 3-6 3-6

-5311.861594505114 3-6 3-7

0.6359149779268591 3-6 3-8

-0.005634811777632651 3-6 3-9

-7.470020328906589e-09 3-7 3-0

6.0086550297353094e-09 3-7 3-1

1.2273120081938105e-08 3-7 3-2

-0.0016759345517555714 3-7 3-3

-0.0036372478853205184 3-7 3-4

0.5522171576708388 3-7 3-5

-5311.86159450474 3-7 3-6

10404.791776495724 3-7 3-7

-5678.617486718309 3-7 3-8

0.7190977572304291 3-7 3-9

-3.549164300069004e-09 3-8 3-0

-1.7187958046720135e-11 3-8 3-1

1.682893460599435e-08 3-8 3-2

1.554309654060553e-08 3-8 3-3

0.0023701421165520913 3-8 3-4

-0.004600820575145942 3-8 3-5

0.6359149777449602 3-8 3-6

-5678.617486718093 3-8 3-7

11792.097345854629 3-8 3-8

-6023.081892914194 3-8 3-9

6.610536347962929e-09 3-9 3-0

2.0549529510724007e-09 3-9 3-1

-1.8822006351580947e-08 3-9 3-2

-2.0845644514126512e-08 3-9 3-3

-2.250921661226498e-08 3-9 3-4

0.003179861094299008 3-9 3-5

-0.005634811778402761 3-9 3-6

0.7190977573104647 3-9 3-7

-6023.081892914879 3-9 3-8

13179.402915035265 3-9 3-9

1128.655011393962 4-0 4-0

-751.2828855015545 4-0 4-1

-66.78433280520503 4-0 4-2

8.070882142068228 4-0 4-3

-1.1749041754695693 4-0 4-4

0.1933211901484433 4-0 4-5

-0.03474078278970283 4-0 4-6

0.0066872562478653 4-0 4-7

-0.001362693029976065 4-0 4-8

0.0002917135931918137 4-0 4-9

-751.282885501159 4-1 4-0

2129.772561921105 4-1 4-1

-1057.601613893642 4-1 4-2

115.12256504875802 4-1 4-3

-16.057600725028806 4-1 4-4

2.6133267126020243 4-1 4-5

-0.4712550388625312 4-1 4-6

0.09155117376807143 4-1 4-7

-0.018861391745732675 4-1 4-8

0.004082225306329401 4-1 4-9

-66.78433280503208 4-2 4-0

-1057.601613893709 4-2 4-1

3124.2641087577395 4-2 4-2

1289.3028478850042 4-2 4-3

-162.02904977860078 4-2 4-4

25.25658496028447 4-2 4-5

-4.502486020760365 4-2 4-6

0.8773619107148107 4-2 4-7

-0.18237001156364174 4-2 4-8

0.039897214642924506 4-2 4-9

8.070882142345539 4-3 4-0

115.12256504848972 4-3 4-1

1289.3028478852045 4-3 4-2

4112.129848308045 4-3 4-3

1481.8176911609225 4-3 4-4

-208.17408394809536 4-3 4-5

35.531032448205565 4-3 4-6

-6.84117280998916 4-3 4-7

1.4257378742573898 4-3 4-8

-0.31459932101800936 4-3 4-9

-1.174904175603194 4-4 4-0

-16.057600724820247 4-4 4-1

-162.02904977832975 4-4 4-2

1481.817691160767 4-4 4-3

5093.369978469004 4-4 4-4

1648.9335620784314 4-4 4-5

-253.7309472201032 4-4 4-6

46.7561994133784 4-4 4-7

-9.62336793775772 4-4 4-8

2.1281156933462775 4-4 4-9

0.19332119003370307 4-5 4-0

2.6133267124731 4-5 4-1

25.256584960078726 4-5 4-2

-208.1740839482335 4-5 4-3

1648.9335620781849 4-5 4-4

6067.984699016189 4-5 4-5

1797.751711366838 4-5 4-6

-298.7652758216716 4-5 4-7

58.83091484150714 4-5 4-8

-12.842115892268453 4-5 4-9

-0.03474078247627055 4-6 4-0

-0.4712550390017558 4-6 4-1

-4.502486020924174 4-6 4-2

35.53103244819991 4-6 4-3

-253.73094722024632 4-6 4-4

1797.7517113673143 4-6 4-5

7035.974211026156 4-6 4-6

1932.5076648096135 4-6 4-7

-343.3076012188103 4-6 4-8

71.6723428603508 4-6 4-9

0.006687256167202796 4-7 4-0

0.09155117428806214 4-7 4-1

0.8773619109545638 4-7 4-2

-6.841172809646338 4-7 4-3

46.75619941341097 4-7 4-4

-298.7652758217366 4-7 4-5

1932.5076648092945 4-7 4-6

7997.338716911181 4-7 4-7

2055.9717015118604 4-7 4-8

-387.37411738537617 4-7 4-9

-0.001362693343760369 4-8 4-0

-0.018861392045199793 4-8 4-1

-0.18237001132844544 4-8 4-2

1.4257378743191431 4-8 4-3

-9.623367937310192 4-8 4-4

58.83091484107797 4-8 4-5

-343.3076012186875 4-8 4-6

2055.971701511691 4-8 4-7

8952.078405128808 4-8 4-8

2170.0750822801706 4-8 4-9

0.000291713409212514 4-9 4-0

0.0040822253701117625 4-9 4-1

0.03989721449574135 4-9 4-2

-0.31459932094539633 4-9 4-3

2.1281156931170564 4-9 4-4

-12.842115892232357 4-9 4-5

71.67234286031237 4-9 4-6

-387.37411738513674 4-9 4-7

2170.0750822802747 4-9 4-8

9900.193244883289 4-9 4-9

1263.4534199615948 5-0 5-0

-868.3588682833474 5-0 5-1

18.563644500670193 5-0 5-2

0.33265627314856516 5-0 5-3

-0.08361818748432626 5-0 5-4

0.008465367534381485 5-0 5-5

-0.0007372010912137499 5-0 5-6

6.180536196750855e-05 5-0 5-7

-5.166730738248866e-06 5-0 5-8

4.34134715865957e-07 5-0 5-9

-868.3588682840038 5-1 5-0

2857.2137150391072 5-1 5-1

-1226.3065827401153 5-1 5-2

32.19140273633582 5-1 5-3

0.657546711029994 5-1 5-4

-0.18629195471980786 5-1 5-5

0.020685026871369416 5-1 5-6

-0.0019469121372356832 5-1 5-7

0.00017456866005267566 5-1 5-8

-1.5510008996644242e-05 5-1 5-9

18.563644500256714 5-2 5-0

-1226.3065827400455 5-2 5-1

4449.377796742694 5-2 5-2

-1499.7824110925799 5-2 5-3

45.5792868374931 5-2 5-4

1.0274128709237687 5-2 5-5

-0.32148315850608533 5-2 5-6

0.03860307910254546 5-2 5-7

-0.0038867964994953874 5-2 5-8

0.00036984988237421385 5-2 5-9

0.33265627372132656 5-3 5-0

32.19140273696257 5-3 5-1

-1499.7824110927295 5-3 5-2

6039.945671752885 5-3 5-3

-1729.3382097615636 5-3 5-4

58.91164268910006 5-3 5-5

1.435670293502568 5-3 5-6

-0.48926631660100384 5-3 5-7

0.062883378764095 5-3 5-8

-0.006719899006386948 5-3 5-9

-0.0836181869954098 5-4 5-0

0.6575467110906317 5-4 5-1

45.57928683733121 5-4 5-2

-1729.3382097612364 5-4 5-3

7628.917346774077 5-4 5-4

-1930.7053478479581 5-4 5-5

72.23582057234125 5-4 5-6

1.8763489581178874 5-4 5-7

-0.6893727729106089 5-4 5-8

0.09409227554234434 5-4 5-9

0.00846536738312173 5-5 5-0

-0.18629195500527107 5-5 5-1

1.0274128710893535 5-5 5-2

58.91164268936336 5-5 5-3

-1930.7053478476732 5-5 5-4

9216.292828357513 5-5 5-5

-2111.963546083432 5-5 5-6

85.56952142647151 5-5 5-7

2.344538611078576 5-5 5-8

-0.9214659515276793 5-5 5-9

-0.0007372010971974322 5-6 5-0

0.020685027015200363 5-6 5-1

-0.32148315868896304 5-6 5-2

1.4356702930844563 5-6 5-3

72.2358205717951 5-6 5-4

-2111.9635460840605 5-6 5-5

10802.072123246522 5-6 5-6

-2277.9193503218985 5-6 5-7

98.92079143837282 5-6 5-8

2.83615160322978 5-6 5-9

6.1805284469885e-05 5-7 5-0

-0.001946911879906324 5-7 5-1

0.03860307884415182 5-7 5-2

-0.48926631625827 5-7 5-3

1.876348958480719 5-7 5-4

85.56952142626551 5-7 5-5

-2277.9193503220104 5-7 5-6

12386.255238136682 5-7 5-7

-2431.7088840366714 5-7 5-8

112.29374692039482 5-7 5-9

-5.166834578672702e-06 5-8 5-0

0.00017456866369947163 5-8 5-1

-0.0038867964708194647 5-8 5-2

0.06288337896457463 5-8 5-3

-0.6893727726758172 5-8 5-4

2.344538611081532 5-8 5-5

98.92079143852561 5-8 5-6

-2431.708884036928 5-8 5-7

13968.84218034822 5-8 5-8

-2575.5135332107416 5-8 5-9

4.3411726278950834e-07 5-9 5-0

-1.5510094370760574e-05 5-9 5-1

0.0003698504080026036 5-9 5-2

-0.006719898876838992 5-9 5-3

0.09409227526845676 5-9 5-4

-0.921465951504743 5-9 5-5

2.836151602948007 5-9 5-6

112.29374692021611 5-9 5-7

-2575.5135332103982 5-9 5-8

15549.83296479995 5-9 5-9

1055.1249868601396 6-0 6-0

-563.9074882258618 6-0 6-1

-75.43441770357208 6-0 6-2

9.235797198729571 6-0 6-3

-0.8043656339259675 6-0 6-4

0.06533498739142135 6-0 6-5

-0.005280448189317114 6-0 6-6

0.000434297618139814 6-0 6-7

-3.666598213808294e-05 6-0 6-8

3.19475946718997e-06 6-0 6-9

-563.9074882251751 6-1 6-0

2769.5624460267213 6-1 6-1

-797.7527559478384 6-1 6-2

-130.11526029564925 6-1 6-3

18.41955398807633 6-1 6-4

-1.7946583149999178 6-1 6-5

0.15975900603925364 6-1 6-6

-0.013952012451006565 6-1 6-7

0.0012272024296588755 6-1 6-8

-0.00010995762903986359 6-1 6-9

-75.43441770369509 6-2 6-0

-797.7527559478646 6-2 6-1

4482.135501279694 6-2 6-2

-977.3593565503033 6-2 6-3

-183.24705488372183 6-2 6-4

29.041661700234727 6-2 6-5

-3.101585730818 6-2 6-6

0.298360788462819 6-2 6-7

-0.027866558635738767 6-2 6-8

0.002600746173310785 6-2 6-9

9.235797198509829 6-3 6-0

-130.11526029577317 6-3 6-1

-977.359356551073 6-3 6-2

6192.84423302175 6-3 6-3

-1128.9088107569282 6-3 6-4

-235.5867354889656 6-3 6-5

40.9549520479313 6-3 6-6

-4.727283982554159 6-3 6-7

0.4863700916666889 6-3 6-8

-0.04820126674565581 6-3 6-9

-0.804365633993112 6-4 6-0

18.419553987874636 6-4 6-1

-183.24705488342602 6-4 6-2

-1128.9088107568864 6-4 6-3

7901.688721647262 6-4 6-4

-1262.5366203040317 6-4 6-5

-287.3304047784627 6-4 6-6

54.02478080718345 6-4 6-7

-6.67059094251487 6-4 6-8

0.7282781346103118 6-4 6-9

0.06533498687020672 6-5 6-0

-1.7946583153125282 6-5 6-1

29.041661700104726 6-5 6-2

-235.58673548960587 6-5 6-3

-1262.5366203036806 6-5 6-4

9608.669047807278 6-5 6-5

-1383.437759874263 6-5 6-6

-338.55276707122766 6-5 6-7

68.14251679270382 6-5 6-8

-8.92968805588432 6-5 6-9

-0.0052804479540263655 6-6 6-0

0.15975900620741246 6-6 6-1

-3.101585730946603 6-6 6-2

40.954952047871444 6-6 6-3

-287.3304047791007 6-6 6-4

-1383.4377598739698 6-6 6-5

11313.785292073546 6-6 6-6

-1494.6962676571222 6-6 6-7

-389.28899348422783 6-6 6-8

83.21977817920953 6-6 6-9

0.0004342978323764279 6-7 6-0

-0.013952011859316424 6-7 6-1

0.29836078833610324 6-7 6-2

-4.727283982554525 6-7 6-3

54.02478080743333 6-7 6-4

-338.55276707135863 6-7 6-5

-1494.6962676566354 6-7 6-6

13017.037535166719 6-7 6-7

-1598.3201439341974 6-7 6-8

-439.55816481697275 6-7 6-9

-3.6665644039313906e-05 6-8 6-0

0.0012272024470478173 6-8 6-1

-0.02786655827699422 6-8 6-2

0.4863700919766334 6-8 6-3

-6.670590942528541 6-8 6-4

68.14251679292539 6-8 6-5

-389.2889934840782 6-8 6-6

-1598.3201439339225 6-8 6-7

14718.425858339411 6-8 6-8

-1695.7030866183502 6-8 6-9

3.1940623698327586e-06 6-9 6-0

-0.00010995747496834785 6-9 6-1

0.0026007457976971998 6-9 6-2

-0.04820126688734816 6-9 6-3

0.7282781341444964 6-9 6-4

-8.92968805661772 6-9 6-5

83.21977817932179 6-9 6-6

-439.55816481680813 6-9 6-7

-1695.703086618569 6-9 6-8

16417.9503441647 6-9 6-9

EL_ST 1 1

9517.5614 0 0

197.61038653184391 1-0 1-0

34.22212466324126 1-0 1-1

0.03479535215288365 1-0 1-2

-1.047788719653239e-05 1-0 1-3

8.070080680498619e-05 1-0 1-4

2.5660355028717107e-09 1-0 1-5

2.042963628045881e-09 1-0 1-6

-1.9764603068483934e-09 1-0 1-7

-1.3244559390628719e-09 1-0 1-8

1.6572827505390022e-09 1-0 1-9

34.22212466326654 1-1 1-0

592.8311595691818 1-1 1-1

48.39738072741335 1-1 1-2

-0.060105947684945704 1-1 1-3

2.09612938730839e-05 1-1 1-4

0.0001804565122011287 1-1 1-5

1.0420142723194248e-09 1-1 1-6

1.561531482946161e-09 1-1 1-7

-3.6001953329672894e-10 1-1 1-8

8.177790553062458e-10 1-1 1-9

0.034795352202309004 1-2 1-0

48.3973807274224 1-2 1-1

988.0519325593536 1-2 1-2

-59.27442901803229 1-2 1-3

0.08477444234711129 1-2 1-4

3.3142500888070565e-05 1-2 1-5

0.00031256002409671017 1-2 1-6

2.6175767537245385e-09 1-2 1-7

6.104177123681136e-09 1-2 1-8

-4.86259018633305e-09 1-2 1-9

-1.0477953860290485e-05 1-3 1-0

-0.06010594759331411 1-3 1-1

-59.274429018208956 1-3 1-2

1383.2727054981615 1-3 1-3

-68.4441979879376 1-3 1-4

-0.10914872318613789 1-3 1-5

-4.687352936266614e-05 1-3 1-6

-0.00047744983260244805 1-3 1-7

4.040153430173148e-09 1-3 1-8

-5.615891606467721e-09 1-3 1-9

8.070069664315078e-05 1-4 1-0

2.0961300655837034e-05 1-4 1-1

0.084774442300386 1-4 1-2

-68.44419798788182 1-4 1-3

1778.4934783881504 1-4 1-4

76.52292055698456 1-4 1-5

0.13331852640828856 1-4 1-6

6.199999857880965e-05 1-4 1-7

0.0006752160572523167 1-4 1-8

-4.43325496157687e-09 1-4 1-9

2.44630705628808e-09 1-5 1-0

0.00018045648095359225 1-5 1-1

3.314262941863904e-05 1-5 1-2

-0.10914872317243862 1-5 1-3

76.52292055703954 1-5 1-4

2173.714251221954 1-5 1-5

83.8266385460272 1-5 1-6

0.1573176837622441 1-5 1-7

7.842238421524805e-05 1-5 1-8

0.0009058932052692728 1-5 1-9

2.009780103267864e-09 1-6 1-0

9.027333597640131e-10 1-6 1-1

0.00031255994765562835 1-6 1-2

-4.687350549348301e-05 1-6 1-3

0.13331852644955688 1-6 1-4

83.82663854616976 1-6 1-5

2568.9350240097056 1-6 1-6

90.54309536473805 1-6 1-7

0.18116184175744365 1-6 1-8

9.604164078049195e-05 1-6 1-9

-1.9092506564311746e-09 1-7 1-0

1.6132973317633643e-09 1-7 1-1

2.4354010750665686e-09 1-7 1-2

-0.00047744972751621683 1-7 1-3

6.200000227231682e-05 1-7 1-4

0.15731768384205225 1-7 1-5

90.5430953648443 1-7 1-6

2964.155796743439 1-7 1-7

96.79461624855848 1-7 1-8

0.20485925324919663 1-7 1-9

-1.2652019921966537e-09 1-8 1-0

-2.3919839802839063e-10 1-8 1-1

6.054568039961275e-09 1-8 1-2

3.987250387268976e-09 1-8 1-3

0.0006752160321089487 1-8 1-4

7.842233882312615e-05 1-8 1-5

0.18116184170571614 1-8 1-6

96.79461624862033 1-8 1-7

3359.376569429267 1-8 1-8

102.66616862381443 1-8 1-9

1.7123384062575632e-09 1-9 1-0

8.823630339186258e-10 1-9 1-1

-4.846695420680909e-09 1-9 1-2

-5.672894420934169e-09 1-9 1-3

-4.4166161965633045e-09 1-9 1-4

0.0009058932324713695 1-9 1-5

9.604164056110035e-05 1-9 1-6

0.20485925327204768 1-9 1-7

102.66616862361947 1-9 1-8

3754.5973420636883 1-9 1-9

322.62920250190325 2-0 2-0

570.3687443839437 2-0 2-1

0.056808741075855096 2-0 2-2

-0.00017466034666588267 2-0 2-3

0.0001317554480350465 2-0 2-4

3.82383580928014e-09 2-0 2-5

3.718804723210067e-09 2-0 2-6

-3.041126716052389e-09 2-0 2-7

-2.378052647727947e-09 2-0 2-8

2.5607242277736494e-09 2-0 2-9

570.368744383985 2-1 2-0

967.8876074486633 2-1 2-1

806.623012166777 2-1 2-2

-0.0981321547387779 2-1 2-3

0.0003493268593964518 2-1 2-4

0.00029462289835788805 2-1 2-5

1.7863740014778532e-09 2-1 2-6

2.4659101082062354e-09 2-1 2-7

-7.866443862747313e-10 2-1 2-8

1.5871848049000744e-09 2-1 2-9

0.05680874115651591 2-2 2-0

806.6230121667918 2-2 2-1

1613.1460123540976 2-2 2-2

-987.9071502951426 2-2 2-3

0.13840724968929408 2-2 2-4

0.0005523345114600832 2-2 2-5

0.000510301114234861 2-2 2-6

3.37302260233764e-09 2-2 2-7

1.1035697641742432e-08 2-2 2-8

-7.50147677353025e-09 2-2 2-9

-0.00017466045549307068 2-3 2-0

-0.09813215458916602 2-3 2-1

-987.907150295431 2-3 2-2

2258.4044171513387 2-3 2-3

-1140.7366330929108 2-3 2-4

-0.178201986121735 2-3 2-5

-0.0007811185599863271 2-3 2-6

-0.0007795126945902021 2-3 2-7

6.222084247431126e-09 2-3 2-8

-8.943302931322416e-09 2-3 2-9

0.00013175526819319953 2-4 2-0

0.0003493268704645341 2-4 2-1

0.1384072496130102 2-4 2-2

-1140.7366330928196 2-4 2-3

2903.662821898234 2-4 2-4

1275.3820091442115 2-4 2-5

0.21766290930520427 2-4 2-6

0.0010333126721983163 2-4 2-7

0.0011023945192519553 2-4 2-8

-5.5438862545498e-09 2-4 2-9

3.6283614469889696e-09 2-5 2-0

0.00029462284730147966 2-5 2-1

0.0005523347213059092 2-5 2-2

-0.17820198609933868 2-5 2-3

1275.3820091443013 2-5 2-4

3548.92122646571 2-5 2-5

1397.1106423553774 2-5 2-6

0.2568452023954251 2-5 2-7

0.001307049539785603 2-5 2-8

0.0014790114976431356 2-5 2-9

3.6646201082020377e-09 2-6 2-0

1.5589678927008247e-09 2-6 2-1

0.0005103009894162737 2-6 2-2

-0.000781118521079456 2-6 2-3

0.21766290937262056 2-6 2-4

1397.1106423556103 2-6 2-5

4194.179631027655 2-6 2-6

1509.051589357611 2-6 2-7

0.29577444965502764 2-6 2-8

0.0016007831993913777 2-6 2-9

-2.931410232870357e-09 2-7 2-0

2.5504357853463156e-09 2-7 2-1

3.075567111343615e-09 2-7 2-2

-0.0007795125230779785 2-7 2-3

0.0010333126782233117 2-7 2-4

0.2568452025257102 2-7 2-5

1509.0515893577845 2-7 2-6

4839.438035457946 2-7 2-7

1613.2436041755768 2-7 2-8

0.3344640941149919 2-7 2-9

-2.2813057096383194e-09 2-8 2-0

-5.8937342669873e-10 2-8 2-1

1.095466758344933e-08 2-8 2-2

6.1357029419447985e-09 2-8 2-3

0.001102394478211564 2-8 2-4

0.001307049465690227 2-8 2-5

0.29577444957044463 2-8 2-6

1613.2436041756778 2-8 2-7

5484.696439859632 2-8 2-8

1711.1028104735753 2-8 2-9

2.6506087695166175e-09 2-9 2-0

1.6926295760053004e-09 2-9 2-1

-7.47553572443255e-09 2-9 2-2

-9.036393511098685e-09 2-9 2-3

-5.516737201144406e-09 2-9 2-4

0.0014790115420257202 2-9 2-5

0.0016007831989905549 2-9 2-6

0.3344640941522812 2-9 2-7

1711.1028104732573 2-9 2-8

6129.95484417686 2-9 2-9

693.6527853757384 3-0 3-0

-621.7019313772987 3-0 3-1

0.12213878301514569 3-0 3-2

0.00019038994416729687 3-0 3-3

0.00028327770178918337 3-0 3-4

9.53448521922473e-09 3-0 3-5

6.618480223048866e-09 3-0 3-6

-7.205594357224675e-09 3-0 3-7

-4.338163248424344e-09 3-0 3-8

6.0265304319573305e-09 3-0 3-9

-621.7019313772099 3-1 3-0

2080.9583560549454 3-1 3-1

-879.2190832769944 3-1 3-2

-0.21098414973835133 3-1 3-3

-0.00038075641127584286 3-1 3-4

0.0006334391549798891 3-1 3-5

3.5349881173174495e-09 3-1 3-6

5.601756673092704e-09 3-1 3-7

-9.7699336055413e-10 3-1 3-8

2.5071780487761133e-09 3-1 3-9

0.12213878318857496 3-2 3-0

-879.2190832769627 3-2 3-1

3468.2639265171442 3-2 3-2

1076.8187938198066 3-2 3-3

0.29757559804670564 3-2 3-4

-0.0006020303660140247 3-2 3-5

0.0010971508651575965 3-2 3-6

1.0486634087814012e-08 3-2 3-7

1.9884725009315497e-08 3-2 3-8

-1.7699335731325047e-08 3-2 3-9

0.00019038971016752633 3-3 3-0

-0.21098414941684496 3-3 3-1

1076.8187938191866 3-3 3-2

4855.56949683413 3-3 3-3

1243.402930057416 3-3 3-4

-0.38313430937319026 3-3 3-5

0.0008513816068589825 3-3 3-6

-0.0016759423661342559 3-3 3-7

1.4721162298310431e-08 3-3 3-8

-2.0038106609615572e-08 3-3 3-9

0.0002832773150844482 3-4 3-0

-0.00038075638755052016 3-4 3-1

0.2975755978827692 3-4 3-2

1243.4029300576115 3-4 3-3

6242.875066937422 3-4 3-4

-1390.1663899183302 3-4 3-5

0.46797522251108603 3-4 3-6

-0.0011263035510567555 3-4 3-7

0.0023701447811611148 3-4 3-8

-1.800418346652618e-08 3-4 3-9

9.114221912819019e-09 3-5 3-0

0.000633439045326561 3-5 3-1

-0.0006020299148454986 3-5 3-2

-0.3831343093254418 3-5 3-3

-1390.1663899181372 3-5 3-4

7630.180636969926 3-5 3-5

-1522.8506001397725 3-5 3-6

0.5522171692873599 3-5 3-7

-0.0014246874473475785 3-5 3-8

0.003179866879448924 3-5 3-9

6.502000620260787e-09 3-6 3-0

3.046100510583626e-09 3-6 3-1

0.0010971505968587405 3-6 3-2

0.0008513816905698619 3-6 3-3

0.46797522265615044 3-6 3-4

-1522.850600139272 3-6 3-5

9017.486206740341 3-6 3-6

-1644.8662323805527 3-6 3-7

0.6359150159973979 3-6 3-8

-0.0017448850678295025 3-6 3-9

-6.96967567430956e-09 3-7 3-0

5.783503576698167e-09 3-7 3-1

9.847114019232424e-09 3-7 3-2

-0.0016759419971740753 3-7 3-3

-0.0011263035380419087 3-7 3-4

0.552217169567939 3-7 3-5

-1644.8662323801796 3-7 3-6

10404.791776383809 3-7 3-7

-1758.4355285629667 3-7 3-8

0.7190977765662865 3-7 3-9

-4.130153778817965e-09 3-8 3-0

-5.529274108472045e-10 3-8 3-1

1.9710510472803166e-08 3-8 3-2

1.4535358647472007e-08 3-8 3-3

0.0023701446927810992 3-8 3-4

-0.00142468760668319 3-8 3-5

0.6359150158154989 3-8 3-6

-1758.4355285627498 3-8 3-7

11792.097345787592 3-8 3-8

-1865.1020634431732 3-8 3-9

6.2197929103569e-09 3-9 3-0

2.7338809759832754e-09 3-9 3-1

-1.764356056618139e-08 3-9 3-2

-2.023823705646199e-08 3-9 3-3

-1.794581628796473e-08 3-9 3-4

0.003179866974814638 3-9 3-5

-0.0017448850686280337 3-9 3-6

0.7190977766467768 3-9 3-7

-1865.1020634438578 3-9 3-8

13179.402915011686 3-9 3-9

811.8832853097801 4-0 4-0

-537.0007371389806 4-0 4-1

-54.43123965023784 4-0 4-2

7.0141871900354005 4-0 4-3

-1.0448118051312267 4-0 4-4

0.17220540108643823 4-0 4-5

-0.030689774332461228 4-0 4-6

0.005833684628101118 4-0 4-7

-0.0011722265985351261 4-0 4-8

0.0002474286303501129 4-0 4-9

-537.0007371385852 4-1 4-0

1777.3955556055396 4-1 4-1

-754.7963838951594 4-1 4-2

93.67696660823088 4-1 4-3

-13.940695959437917 4-1 4-4

2.3230538394795666 4-1 4-5

-0.4198345121176805 4-1 4-6

0.08091551804544594 4-1 4-7

-0.016466067087093222 4-1 4-8

0.003514816226859341 4-1 4-9

-54.43123965006487 4-2 4-0

-754.7963838952264 4-2 4-1

2736.109374880134 4-2 4-2

918.7496839345158 4-2 4-3

-131.6305333721915 4-2 4-4

21.903609012813405 4-2 4-5

-4.00072183057425 4-2 4-6

0.7817150989787507 4-2 4-7

-0.1612614088751072 4-2 4-8

0.0348559918721304 4-2 4-9

7.014187190312697 4-3 4-0

93.67696660796258 4-3 4-1

918.7496839347162 4-3 4-2

3688.0247982393985 4-3 4-3

1054.3136560035832 4-3 4-4

-168.8396501497798 4-3 4-5

30.78050830411587 4-3 4-6

-6.0761323315662 4-3 4-7

1.2704265468680305 4-3 4-8

-0.2783165697111438 4-3 4-9

-1.044811805264855 4-4 4-0

-13.940695959229387 4-4 4-1

-131.63053337192048 4-4 4-2

1054.3136560034277 4-4 4-3

4633.141881140043 4-4 4-4

1171.410887638278 4-4 4-5

-205.44607259358327 4-4 4-6

40.459786998737286 4-4 4-7

-8.543277005855757 4-4 4-8

1.8964333641912994 4-4 4-9

0.17220540097164827 4-5 4-0

2.323053839350642 4-5 4-1

21.90360901260766 4-5 4-2

-168.83965014991804 4-5 4-3

1171.4108876380312 4-5 4-4

5571.460679654668 4-5 4-5

1275.1612428796868 4-5 4-6

-241.50365381483562 4-5 4-7

50.85056734869653 4-5 4-8

-11.395268351019311 4-5 4-9

-0.030689774019014737 4-6 4-0

-0.41983451225691937 4-6 4-1

-4.000721830738044 4-6 4-2

30.780508304110356 4-6 4-3

-205.44607259372629 4-6 4-4

1275.1612428801636 4-6 4-5

6502.981250051093 4-6 4-6

1368.6240649275228 4-6 4-7

-277.03759993497624 4-6 4-8

61.87808354614998 4-6 4-9

0.005833684547424403 4-7 4-0

0.08091551856543755 4-7 4-1

0.7817150992184967 4-7 4-2

-6.0761323312234765 4-7 4-3

40.45978699876986 4-7 4-4

-241.50365381490064 4-7 4-5

1368.6240649272036 4-7 4-6

7427.703648848012 4-7 4-7

1453.8041280070129 4-7 4-8

-312.0614365863066 4-7 4-9

-0.0011722269123212066 4-8 4-0

-0.016466067386530142 4-8 4-1

-0.16126140863990912 4-8 4-2

1.2704265469298228 4-8 4-3

-8.543277005408257 4-8 4-4

50.850567348267305 4-8 4-5

-277.037599934853 4-8 4-6

1453.8041280068437 4-8 4-7

8345.627922733607 4-8 4-8

1532.1017709490798 4-8 4-9

0.0002474284463512733 4-9 4-0

0.003514816290670124 4-9 4-1

0.03485599172494547 4-9 4-2

-0.2783165696385641 4-9 4-3

1.8964333639620357 4-9 4-4

-11.395268350983159 4-9 4-5

61.87808354611133 4-9 4-6

-312.06143658606743 4-9 4-7

1532.101770949184 4-9 4-8

9256.75397452296 4-9 4-9

858.464494298139 5-0 5-0

343.3080870533259 5-0 5-1

-91.25212644982764 5-0 5-2

7.979736718764265 5-0 5-3

-0.5970507393632083 5-0 5-4

0.04351895036191572 5-0 5-5

-0.0032108054469136265 5-0 5-6

0.0002431205532624481 5-0 5-7

-1.8983149078333484e-05 5-0 5-8

1.5282582711779152e-06 5-0 5-9

343.30808705266946 5-1 5-0

2406.894189576802 5-1 5-1

483.6288830679859 5-1 5-2

-157.6433359431236 5-1 5-3

15.926230119915786 5-1 5-4

-1.332780151061014 5-1 5-5

0.10645426081992326 5-1 5-6

-0.008486082160033374 5-1 5-7

0.0006871265856460244 5-1 5-8

-5.6949124650217977e-05 5-1 5-9

-91.25212645024106 5-2 5-0

483.62888306805576 5-2 5-1

3953.8115861883884 5-2 5-2

590.0239990240367 5-2 5-3

-222.36224224492116 5-2 5-4

25.129051164816175 5-2 5-5

-2.304518713591273 5-2 5-6

0.19888636796994275 5-2 5-7

-0.01695438257426226 5-2 5-8

0.001456548132030844 5-2 5-9

7.979736719337019 5-3 5-0

-157.64333594249683 5-3 5-1

590.0239990238871 5-3 5-2

5499.216711908099 5-3 5-3

678.6556819531536 5-3 5-4

-286.3217111454396 5-3 5-5

35.46360297613728 5-3 5-6

-3.514216861286563 5-3 5-7

0.3243368726399467 5-3 5-8

-0.02933496337132524 5-3 5-9

-0.5970507388743096 5-4 5-0

15.926230119976424 5-4 5-1

-222.36224224508305 5-4 5-2

678.6556819534808 5-4 5-3

7043.109594553427 5-4 5-4

755.8116376992223 5-4 5-5

-349.7576313971099 5-4 5-6

46.815785401892924 5-4 5-7

-4.961377808917909 5-4 5-8

0.4858402124305776 5-4 5-9

0.043518950210641755 5-5 5-0

-1.3327801513465056 5-5 5-1

25.129051164981746 5-5 5-2

-286.32171114517655 5-5 5-3

755.8116376995072 5-5 5-4

8585.490262018093 5-5 5-5

824.7302388589364 5-5 5-6

-412.7593591074201 5-5 5-7

59.09372949583184 5-5 5-8

-6.645017281237717 5-5 5-9

-0.003210805452897309 5-6 5-0

0.10645426096375776 5-6 5-1

-2.304518713774165 5-6 5-2

35.4636029757192 5-6 5-3

-349.75763139765604 5-6 5-4

824.7302388583084 5-6 5-5

10126.358742153529 5-6 5-6

887.3433179350449 5-6 5-7

-475.3685328618076 5-6 5-8

72.22287665286598 5-6 5-9

0.0002431204757506137 5-7 5-0

-0.008486081902714673 5-7 5-1

0.19888636771154555 5-7 5-2

-3.5142168609438507 5-7 5-3

46.81578540225564 5-7 5-4

-412.7593591076261 5-7 5-5

887.3433179349329 5-7 5-6

11665.71506292804 5-7 5-7

944.9148714284449 5-7 5-8

-537.6073695887162 5-7 5-9

-1.898325292408639e-05 5-8 5-0

0.0006871265892892677 5-8 5-1

-0.016954382545607653 5-8 5-2

0.3243368728404903 5-8 5-3

-4.961377808683174 5-8 5-4

59.09372949583485 5-8 5-5

-475.3685328616557 5-8 5-6

944.9148714281886 5-8 5-7

13203.55925233681 5-8 5-8

998.3265896823389 5-8 5-9

1.5282408287596076e-06 5-9 5-0

-5.69492100163407e-05 5-9 5-1

0.001456548657630812 5-9 5-2

-0.029334963241787942 5-9 5-3

0.48584021215654793 5-9 5-4

-6.6450172812147805 5-9 5-5

72.22287665258443 5-9 5-6

-537.6073695888949 5-9 5-7

998.3265896826824 5-9 5-8

14739.891338335485 5-9 5-9

1729.5876363791492 6-0 6-0

-1205.9827409227075 6-0 6-1

-34.80091891118093 6-0 6-2

6.804725016964419 6-0 6-3

-0.6530814710972299 6-0 6-4

0.05538779286509882 6-0 6-5

-0.004588828482856969 6-0 6-6

0.0003835918040793996 6-0 6-7

-3.27600773793443e-05 6-0 6-8

2.8795518516256036e-06 6-0 6-9

-1205.9827409220209 6-1 6-0

3439.226286858624 6-1 6-1

-1703.9772081950255 6-1 6-2

-59.851578185551716 6-1 6-3

13.563877471989528 6-1 6-4

-1.4567241889591696 6-1 6-5

0.1354112110465201 6-1 6-6

-0.01212294860633982 6-1 6-7

0.0010838033265958289 6-1 6-8

-9.823877464520362e-05 6-1 6-9

-34.80091891130394 6-2 6-0

-1703.9772081950514 6-2 6-1

5147.009834112667 6-2 6-2

-2085.0413756748444 6-2 6-3

-84.04265377184538 6-2 6-4

21.37439236917703 6-2 6-5

-2.516869133181883 6-2 6-6

0.2528435051604736 6-2 6-7

-0.024210019188440055 6-2 6-8

0.002296594605672485 6-2 6-9

6.804725016744676 6-3 6-0

-59.85157818567541 6-3 6-1

-2085.0413756756143 6-3 6-2

6852.938359005037 6-3 6-3

-2405.3976563969977 6-3 6-4

-107.72537310006237 6-3 6-5

30.126266192106186 6-3 6-6

-3.835035722849611 6-3 6-7

0.41209510623313106 6-3 6-8

-0.04187071222325919 6-3 6-9

-0.6530814711643744 6-4 6-0

13.563877471787805 6-4 6-1

-84.04265377154957 6-4 6-2

-2405.3976563969563 6-4 6-3

8557.011942244393 6-4 6-4

-2686.843026271053 6-4 6-5

-130.9911837449049 6-4 6-6

39.71892368670575 6-4 6-7

-5.410065094061046 6-4 6-8

0.6169474275939191 6-4 6-9

0.05538779234388064 6-5 6-0

-1.456724189271723 6-5 6-1

21.374392369047115 6-5 6-2

-107.7253731007022 6-5 6-3

-2686.843026270702 6-5 6-4

10259.230664976327 6-5 6-5

-2940.565678822174 6-5 6-6

-153.87529589342967 6-5 6-7

50.071106634173304 6-5 6-8

-7.240265811966616 6-5 6-9

-0.004588828247583984 6-6 6-0

0.13541121121462385 6-6 6-1

-2.5168691333103865 6-6 6-2

30.126266192046558 6-6 6-3

-130.99118374554337 6-6 6-4

-2940.5656788218807 6-6 6-5

11959.594608053143 6-6 6-6

-3173.217226566986 6-6 6-7

-176.39460564672845 6-6 6-8

61.11659094217441 6-6 6-9

0.0003835920183071317 6-7 6-0

-0.012122948014596388 6-7 6-1

0.2528435050336939 6-7 6-2

-3.8350357228500442 6-7 6-3

39.71892368695569 6-7 6-4

-153.87529589356063 6-7 6-5

-3173.217226566499 6-7 6-6

13658.10385248702 6-7 6-7

-3389.1348912457697 6-7 6-8

-198.55855538813194 6-7 6-9

-3.275973926636441e-05 6-8 6-0

0.0010838033439883234 6-8 6-1

-0.024210018829667085 6-8 6-2

0.41209510654318926 6-8 6-3

-5.41006509407466 6-8 6-4

50.07110663439488 6-8 6-5

-176.39460564657884 6-8 6-6

-3389.134891245495 6-8 6-7

15354.758480256616 6-8 6-8

-3591.3335472748417 6-8 6-9

2.878854745386608e-06 6-9 6-0

-9.823862055325977e-05 6-9 6-1

0.0022965942300819924 6-9 6-2

-0.0418707123648947 6-9 6-3

0.6169474271281037 6-9 6-4

-7.2402658126999455 6-9 6-5

61.11659094228645 6-9 6-6

-198.55855538796732 6-9 6-7

-3591.3335472750605 6-9 6-8

17049.558575142117 6-9 6-9

EL_ST 0 0

1.5526024910172964e-11 1-0 1-0

8.555531165753601 1-0 1-1

4.767586325726825e-11 1-0 1-2

-2.619952265905808e-06 1-0 1-3

-1.6150469850373383e-11 1-0 1-4

-6.0798189806211475e-12 1-0 1-5

6.374567540490261e-12 1-0 1-6

3.0885857565075856e-12 1-0 1-7

-3.5860481251148713e-12 1-0 1-8

-2.4120231636656492e-12 1-0 1-9

8.555531165753601 1-1 1-0

-1.8666268530864727e-10 1-1 1-1

12.099345182572062 1-1 1-2

7.893508069400923e-11 1-1 1-3

5.2398572177296695e-06 1-1 1-4

3.5360603334311236e-13 1-1 1-5

1.4153822428322811e-12 1-1 1-6

-1.3896661599233084e-12 1-1 1-7

-3.3066622356913357e-12 1-1 1-8

4.1907449732647706e-12 1-1 1-9

4.767586325726825e-11 1-2 1-0

12.099345182572062 1-2 1-1

2.0446755399916583e-10 1-2 1-2

-14.81860725441835 1-2 1-3

-5.191891361278067e-11 1-2 1-4

8.2849516928221e-06 1-2 1-5

-1.606331734294031e-11 1-2 1-6

-1.497542107681252e-11 1-2 1-7

1.7787632478061255e-11 1-2 1-8

7.274047683638815e-12 1-2 1-9

-2.619952265961319e-06 1-3 1-0

7.893508069400923e-11 1-3 1-1

-14.81860725441835 1-3 1-2

1.9096280112762543e-10 1-3 1-3

-17.111049496329507 1-3 1-4

1.8153545333632337e-10 1-3 1-5

-1.1716604217287471e-05 1-3 1-6

-4.596067970652484e-11 1-3 1-7

-6.220288173435262e-12 1-3 1-8

3.749583976642157e-12 1-3 1-9

-1.6150469850373383e-11 1-4 1-0

5.2398572178406105e-06 1-4 1-1

-5.191891361278067e-11 1-4 1-2

-17.111049496329507 1-4 1-3

6.656577511421347e-10 1-4 1-4

19.130730136936968 1-4 1-5

1.5044099299643676e-10 1-4 1-6

1.5499656464433575e-05 1-4 1-7

1.5904833006175068e-11 1-4 1-8

2.816927247026347e-11 1-4 1-9

-6.079680202743069e-12 1-5 1-0

3.5371705564557487e-13 1-5 1-1

8.284951692600056e-06 1-5 1-2

1.815347872025086e-10 1-5 1-3

19.130730136936968 1-5 1-4

-3.178302065975913e-10 1-5 1-5

20.95665963520294 1-5 1-6

7.343636809764575e-11 1-5 1-7

1.9605759064816874e-05 1-5 1-8

3.630029610235397e-11 1-5 1-9

6.374678562792724e-12 1-6 1-0

1.415410106828114e-12 1-6 1-1

-1.6063150809486615e-11 1-6 1-2

-1.1716604217287471e-05 1-6 1-3

1.5044188117485646e-10 1-6 1-4

20.95665963520294 1-6 1-5

-1.4298251471700496e-10 1-6 1-6

22.63577384027507 1-6 1-7

2.3500223989003644e-10 1-6 1-8

2.401189327072562e-05 1-6 1-9

3.088696778810048e-12 1-7 1-0

-1.3893886041671522e-12 1-7 1-1

-1.497542107681252e-11 1-7 1-2

-4.596051317307115e-11 1-7 1-3

1.5499656464114386e-05 1-7 1-4

7.34368121868556e-11 1-7 1-5

22.63577384027507 1-7 1-6

-6.908518201953484e-10 1-7 1-7

24.198654062687304 1-7 1-8

1.1935608057456193e-10 1-7 1-9

-3.5862424141441807e-12 1-8 1-0

-3.3066071582209734e-12 1-8 1-1

1.7787299411153867e-11 1-8 1-2

-6.2209543072500375e-12 1-8 1-3

1.5903722783150442e-11 1-8 1-4

1.9605759064983408e-05 1-8 1-5

2.3500312806845614e-10 1-8 1-6

24.198654062687304 1-8 1-7

-4.1381564841458385e-10 1-8 1-8

25.66654215722852 1-8 1-9

-2.4119666767324627e-12 1-9 1-0

4.190786606628194e-12 1-9 1-1

7.2741587059412775e-12 1-9 1-2

3.749139887432307e-12 1-9 1-3

2.816925859247566e-11 1-9 1-4

3.629985201314412e-11 1-9 1-5

2.401189327072562e-05 1-9 1-6

1.1935608057456193e-10 1-9 1-7

25.66654215722852 1-9 1-8

-1.4554046856574132e-10 1-9 1-9

-1.035029839613344e-10 2-0 2-0

-57.03687443835733 2-0 2-1

-3.178364238465292e-10 2-0 2-2

1.7466348439964326e-05 2-0 2-3

1.0766987301735753e-10 2-0 2-4

4.053009116473591e-11 2-0 2-5

-4.249689489199682e-11 2-0 2-6

-2.0591275212700227e-11 2-0 2-7

2.3908208746092896e-11 2-0 2-8

1.608056049429133e-11 2-0 2-9

-57.03687443835733 2-1 2-0

1.2444232311281667e-09 2-1 2-1

-80.66230121714707 2-1 2-2

-5.262332791744484e-10 2-1 2-3

-3.49323814494585e-05 2-1 2-4

-2.3598900611432327e-12 2-1 2-5

-9.436141104601821e-12 2-1 2-6

9.261702516027981e-12 2-1 2-7

2.2044791339603193e-11 2-1 2-8

-2.7939761615414227e-11 2-1 2-9

-3.178382002033686e-10 2-2 2-0

-80.66230121714707 2-2 2-1

-1.363126500564249e-09 2-2 2-2

98.79071502945564 2-2 2-3

3.461266828708176e-10 2-2 2-4

-5.523301128754904e-05 2-2 2-5

1.070867838848244e-10 2-2 2-6

9.983398829849094e-11 2-2 2-7

-1.185795905911391e-10 2-2 2-8

-4.849302903677205e-11 2-2 2-9

1.746634844040836e-05 2-3 2-0

-5.262386082449666e-10 2-3 2-1

98.79071502945564 2-3 2-2

-1.2730936305160867e-09 2-3 2-3

114.07366330886337 2-3 2-4

-1.210224809256033e-09 2-3 2-5

7.811069477832444e-05 2-3 2-6

3.0639801806842115e-10 2-3 2-7

4.147499010901042e-11 2-3 2-8

-2.4998225711669875e-11 2-3 2-9

1.0766942892814768e-10 2-4 2-0

-3.49323814485701e-05 2-4 2-1

3.461302355844964e-10 2-4 2-2

114.07366330886336 2-4 2-3

-4.437723077899136e-09 2-4 2-4

-127.53820091291311 2-4 2-5

-1.0029399533095784e-09 2-4 2-6

-0.00010333104310172869 2-4 2-7

-1.0602807520854185e-10 2-4 2-8

-1.8779577892813707e-10 2-4 2-9

4.0530313209340835e-11 2-5 2-0

-2.3598900611432327e-12 2-5 2-1

-5.523301128932366e-05 2-5 2-2

-1.2102301383265512e-09 2-5 2-3

-127.53820091291311 2-5 2-4

2.118866859746049e-09 2-5 2-5

-139.71106423468623 2-5 2-6

-4.895905902913e-10 2-5 2-7

-0.00013070506043399988 2-5 2-8

-2.4199886539122417e-10 2-5 2-9

-4.249667284739189e-11 2-6 2-0

-9.436585193811671e-12 2-6 2-1

1.070858957064047e-10 2-6 2-2

7.811069477832444e-05 2-6 2-3

-1.0029399533095784e-09 2-6 2-4

-139.71106423468623 2-6 2-5

9.531930800221744e-10 2-6 2-6

-150.90515893516715 2-6 2-7

-1.56668988893216e-09 2-6 2-8

-0.00016007928847328046 2-6 2-9

-2.0590609295725886e-11 2-7 2-0

9.262146605237831e-12 2-7 2-1

9.983664589485613e-11 2-7 2-2

3.0640157078209995e-10 2-7 2-3

-0.00010333104309817598 2-7 2-4

-4.895870375776212e-10 2-7 2-5

-150.90515893516715 2-7 2-6

4.605688275205466e-09 2-7 2-7

-161.32436041791533 2-7 2-8

-7.957154934956634e-10 2-7 2-9

2.3908097723790434e-11 2-8 2-0

2.2044347250393343e-11 2-8 2-1

-1.185813669479785e-10 2-8 2-2

4.147418519731757e-11 2-8 2-3

-1.0602807520854185e-10 2-8 2-4

-0.0001307050604306692 2-8 2-5

-1.56668988893216e-09 2-8 2-6

-161.32436041791533 2-8 2-7

2.7587603312895226e-09 2-8 2-8

-171.11028104819013 2-8 2-9

1.607982844098447e-11 2-9 2-0

-2.7939206503901914e-11 2-9 2-1

-4.8494597226794335e-11 2-9 2-2

-2.4997337533250175e-11 2-9 2-3

-1.877938915489952e-10 2-9 2-4

-2.4199886539122417e-10 2-9 2-5

-0.0001600792884750568 2-9 2-6

-7.957119407819846e-10 2-9 2-7

-171.11028104819013 2-9 2-8

9.70260316535132e-10 2-9 2-9

2.3805668547538517e-10 3-0 3-0

131.1848112082219 3-0 3-1

7.310276828320639e-10 3-0 3-2

-4.017260141518197e-05 3-0 3-3

-2.476383542671101e-10 3-0 3-4

-9.322487226626208e-11 3-0 3-5

9.774359099878893e-11 3-0 3-6

4.7357335067332894e-11 3-0 3-7

-5.499090072191848e-11 3-0 3-8

-3.698338336965935e-11 3-0 3-9

131.1848112082219 3-1 3-0

-2.86216561562469e-09 3-1 3-1

185.52329279943825 3-1 3-2

1.2103420488074335e-09 3-1 3-3

8.034447732505013e-05 3-1 3-4

5.425881965948065e-12 3-1 3-5

2.1701824365338945e-11 3-1 3-6

-2.1303403485717354e-11 3-1 3-7

-5.0707001097992364e-11 3-1 3-8

6.426215115595824e-11 3-1 3-9

7.310276828320639e-10 3-2 3-0

185.52329279943825 3-2 3-1

3.135198767267866e-09 3-2 3-2

-227.21864456774802 3-2 3-3

-7.961027392866526e-10 3-2 3-4

0.00012703592595587898 3-2 3-5

-2.4629898121020233e-10 3-2 3-6

-2.29618921099292e-10 3-2 3-7

2.7274005276467506e-10 3-2 3-8

1.1153794554634021e-10 3-2 3-9

-4.017260141695833e-05 3-3 3-0

1.2103456015211123e-09 3-3 3-1

-227.21864456774802 3-3 3-2

2.9280613489390817e-09 3-3 3-3

-262.36942561038575 3-3 3-4

2.783544061912835e-09 3-3 3-5

-0.000179654597996598 3-3 3-6

-7.047171735052871e-10 3-3 3-7

-9.538947409744235e-11 3-3 3-8

5.7494897731658057e-11 3-3 3-9

-2.476383542671101e-10 3-4 3-0

8.034447732682649e-05 3-4 3-1

-7.961169501413679e-10 3-4 3-2

-262.36942561038575 3-4 3-3

1.0206733236373111e-08 3-4 3-4

293.33786209970015 3-4 3-5

2.30676278079045e-09 3-4 3-6

0.00023766139912617668 3-4 3-7

2.438600432697058e-10 3-4 3-8

4.319222757244775e-10 3-4 3-9

-9.322575979416048e-11 3-5 3-0

5.4294346796268655e-12 3-5 3-1

0.00012703592595942476 3-5 3-2

2.7835298510581197e-09 3-5 3-3

293.33786209970015 3-5 3-4

-4.873371040048369e-09 3-5 3-5

321.3354477397784 3-5 3-6

1.126046811350534e-09 3-5 3-7

0.00030062163900179684 3-5 3-8

5.565974348087366e-10 3-5 3-9

9.774137055273968e-11 3-6 3-0

2.1703383881743848e-11 3-6 3-1

-2.4629986938862203e-10 3-6 3-2

-0.00017965459798946481 3-6 3-3

2.3067769916451653e-09 3-6 3-4

321.3354477397784 3-6 3-5

-2.1923938220425043e-09 3-6 3-6

347.0818655508844 3-6 3-7

3.603361165005481e-09 3-6 3-8

0.00036818236348246103 3-6 3-9

4.7358667334962444e-11 3-7 3-0

-2.1300738950458253e-11 3-7 3-1

-2.296162565640329e-10 3-7 3-2

-7.047207262189659e-10 3-7 3-3

0.0002376613991298404 3-7 3-4

1.1260397059231764e-09 3-7 3-5

347.0818655508844 3-7 3-6

-1.0593069532660593e-08 3-7 3-7

371.04602896120525 3-7 3-8

1.8301378190699324e-09 3-7 3-9

-5.499178890033818e-11 3-8 3-0

-5.0705224741152964e-11 3-8 3-1

2.7274005276467506e-10 3-8 3-2

-9.538947409744235e-11 3-8 3-3

2.438600432697058e-10 3-8 3-4

0.0003006216390035732 3-8 3-5

3.603361165005481e-09 3-8 3-6

371.04602896120525 3-8 3-7

-6.3451324194829795e-09 3-8 3-8

393.55364641083736 3-8 3-9

-3.69836036795408e-11 3-9 3-0

6.426303933437794e-11 3-9 3-1

1.1153439283266141e-10 3-9 3-2

5.7494897731658057e-11 3-9 3-3

4.319204993676381e-10 3-9 3-4

5.56599211165576e-10 3-9 3-5

0.00036818236348601374 3-9 3-6

1.8301378190699324e-09 3-9 3-7

393.55364641083736 3-9 3-8

-2.2316442027658923e-09 3-9 3-9

EL_ST 1 1

8.79776251849762e-11 1-0 1-0

48.48134327260374 1-0 1-1

2.701625589907053e-10 1-0 1-2

-1.48463961727817e-05 1-0 1-3

-9.15196807227403e-11 1-0 1-4

-3.445107691962476e-11 1-0 1-5

3.612155019538932e-11 1-0 1-6

1.7503327563381256e-11 1-0 1-7

-2.0323631666485653e-11 1-0 1-8

-1.3668166858743344e-11 1-0 1-9

48.48134327260374 1-1 1-0

-1.0577494435892731e-09 1-1 1-1

68.56295603457502 1-1 1-2

4.4729819848043917e-10 1-1 1-3

2.9692524233727608e-05 1-1 1-4

2.0052848270779577e-12 1-1 1-5

8.020930274133011e-12 1-1 1-6

-7.873368623734223e-12 1-1 1-7

-1.873607172186935e-11 1-1 1-8

2.374822560824441e-11 1-1 1-9

2.701616708122856e-10 1-2 1-0

68.56295603457502 1-2 1-1

1.1586642756356014e-09 1-2 1-2

-83.97210777503732 1-2 1-3

-2.942091015256665e-10 1-2 1-4

4.694805959718114e-05 1-2 1-5

-9.102407716454763e-11 1-2 1-6

-8.486070179891561e-11 1-2 1-7

1.0079781453953274e-10 1-2 1-8

4.1215919566198134e-11 1-2 1-9

-1.48463961727817e-05 1-3 1-0

4.4729997483727857e-10 1-3 1-1

-83.9721077750373 1-3 1-2

1.082113953998487e-09 1-3 1-3

-96.96261381253387 1-3 1-4

1.0286900220535244e-09 1-3 1-5

-6.639409056818923e-05 1-3 1-6

-2.60439669830248e-10 1-3 1-7

-3.525096881065486e-11 1-3 1-8

2.124678211146147e-11 1-3 1-9

-9.152012481195015e-11 1-4 1-0

2.9692524233727608e-05 1-4 1-1

-2.942073251688271e-10 1-4 1-2

-96.96261381253389 1-4 1-3

3.772051115902286e-09 1-4 1-4

108.40747077597615 1-4 1-5

8.52494963510253e-10 1-4 1-6

8.783138663531753e-05 1-4 1-7

9.012124380092246e-11 1-4 1-8

1.596254239904246e-10 1-4 1-9

-3.445174305343954e-11 1-5 1-0

2.005728916287808e-12 1-5 1-1

4.6948059593628425e-05 1-5 1-2

1.0286900220535244e-09 1-5 1-3

108.40747077597615 1-5 1-4

-1.801048199467914e-09 1-5 1-5

118.75440459948334 1-5 1-6

4.161506694799755e-10 1-5 1-7

0.00011109930136532498 1-5 1-8

2.0569546066440125e-10 1-5 1-9

3.612177223999424e-11 1-6 1-0

8.02115058401446e-12 1-6 1-1

-9.102363307533778e-11 1-6 1-2

-6.6394090567308e-05 1-6 1-3

8.524931871534136e-10 1-6 1-4

118.75440459948334 1-6 1-5

-8.102389870145998e-10 1-6 1-6

128.26938509489207 1-6 1-7

1.3316796554363464e-09 1-6 1-8

0.00013606739519422817 1-6 1-9

1.750310551877633e-11 1-7 1-0

-7.872480445314523e-12 1-7 1-1

-8.486070179891561e-11 1-7 1-2

-2.6043922574103817e-10 1-7 1-3

8.783138663448486e-05 1-7 1-4

4.161453404094573e-10 1-7 1-5

128.26938509489207 1-7 1-6

-3.9148133623712056e-09 1-7 1-7

137.12570635522803 1-7 1-8

6.763567483858424e-10 1-7 1-9

-2.0322077354251178e-11 1-8 1-0

-1.8736512341632247e-11 1-8 1-1

1.0079603818269334e-10 1-8 1-2

-3.525194025580141e-11 1-8 1-3

9.012213197934216e-11 1-8 1-4

0.00011109930136776747 1-8 1-5

1.3316761027226676e-09 1-8 1-6

137.12570635522803 1-8 1-7

-2.3449402419828402e-09 1-8 1-8

145.44373889096164 1-8 1-9

-1.36682332119163e-11 1-9 1-0

2.3748336630546873e-11 1-9 1-1

4.121747387843261e-11 1-9 1-2

2.124544984383192e-11 1-9 1-3

1.596254239904246e-10 1-9 1-4

2.0569723702124065e-10 1-9 1-5

0.00013606739519778088 1-9 1-6

6.763567483858424e-10 1-9 1-7

145.44373889096164 1-9 1-8

-8.247482696788211e-10 1-9 1-9

4.140066067748194e-11 2-0 2-0

22.81474977534294 2-0 2-1

1.2713607944192518e-10 2-0 2-2

-6.986539375452807e-06 2-0 2-3

-4.306777157125907e-11 2-0 2-4

-1.6212813508170373e-11 2-0 2-5

1.6999068819245622e-11 2-0 2-6

8.235766452494026e-12 2-0 2-7

-9.562961533760017e-12 2-0 2-8

-6.431994043279357e-12 2-0 2-9

22.81474977534294 2-1 2-0

-4.977689371798988e-10 2-1 2-1

32.26492048685883 2-1 2-2

2.1049428866604103e-10 2-1 2-3

1.3972952578984125e-05 2-1 2-4

9.43467526326458e-13 2-1 2-5

3.77489142375233e-12 2-1 2-6

-3.704592188569222e-12 2-1 2-7

-8.818129386411779e-12 2-1 2-8

1.117522741012067e-11 2-1 2-9

1.2713607944192518e-10 2-2 2-0

32.26492048685883 2-2 2-1

5.452580609244251e-10 2-2 2-2

-39.516286011782256 2-2 2-3

-1.3844836388443582e-10 2-2 2-4

2.2093204512714862e-05 2-2 2-5

-4.283551291450749e-11 2-2 2-6

-3.993408034808026e-11 2-2 2-7

4.74319472587581e-11 2-2 2-8

1.9398982420433096e-11 2-2 2-9

-6.986539375674852e-06 2-3 2-0

2.1049384457683118e-10 2-3 2-1

-39.516286011782256 2-3 2-2

5.092299915077092e-10 2-3 2-3

-45.62946532354536 2-3 2-4

4.840909895165169e-10 2-3 2-5

-3.1244277912317875e-05 2-3 2-6

-1.2255862991139566e-10 2-3 2-7

-1.6587745066421742e-11 2-3 2-8

9.999612249345091e-12 2-3 2-9

-4.3067993615864e-11 2-4 2-0

1.3972952579872303e-05 2-4 2-1

-1.3844747570601612e-10 2-4 2-2

-45.62946532354536 2-4 2-3

1.7750867442600793e-09 2-4 2-4

51.015280365165246 2-4 2-5

4.0117509314541167e-10 2-4 2-6

4.133241723841552e-05 2-4 2-7

4.2411185674495755e-11 2-4 2-8

7.511730126830242e-11 2-4 2-9

-1.6212924530472836e-11 2-5 2-0

9.43467526326458e-13 2-5 2-1

2.2093204512713127e-05 2-5 2-2

4.840909895165169e-10 2-5 2-3

51.01528036516525 2-5 2-4

-8.475389279283263e-10 2-5 2-5

55.884425693874505 2-5 2-6

1.958309070460018e-10 2-5 2-7

5.228202417395522e-05 2-5 2-8

9.679812507101815e-11 2-5 2-9

1.6998291663128384e-11 2-6 2-0

3.774835912601098e-12 2-6 2-1

-4.283551291450749e-11 2-6 2-2

-3.1244277911873786e-05 2-6 2-3

4.0117420496699197e-10 2-6 2-4

55.884425693874505 2-6 2-5

-3.8129144286358496e-10 2-6 2-6

60.362063574066866 2-6 2-7

6.266720475878174e-10 2-6 2-8

6.403171538948982e-05 2-6 2-9

8.235877583216706e-12 2-7 2-0

-3.703926054754447e-12 2-7 2-1

-3.9934302392685184e-11 2-7 2-2

-1.2255951808981536e-10 2-7 2-3

4.13324172393037e-05 2-7 2-4

1.958317952244215e-10 2-7 2-5

60.36206357406686 2-7 2-6

-1.8422667835693574e-09 2-7 2-7

64.52974416716614 2-7 2-8

3.182858421268975e-10 2-7 2-9

-9.562961533760017e-12 2-8 2-0

-8.817907341806854e-12 2-8 2-1

4.743239134796795e-11 2-8 2-2

-1.658775894420955e-11 2-8 2-3

4.2412073852915455e-11 2-8 2-4

5.228202417595362e-05 2-8 2-5

6.266720475878174e-10 2-8 2-6

64.52974416716614 2-8 2-7

-1.1035048430585448e-09 2-8 2-8

68.44411241927605 2-8 2-9

-6.432050638632761e-12 2-9 2-0

1.117592129951106e-11 2-9 2-1

1.9397650152803546e-11 2-9 2-2

9.999834293950016e-12 2-9 2-3

7.511652411218519e-11 2-9 2-4

9.679812507101815e-11 2-9 2-5

6.403171538904573e-05 2-9 2-6

3.182858421268975e-10 2-9 2-7

68.44411241927605 2-9 2-8

-3.881126531268819e-10 2-9 2-9

7.245137823019832e-11 3-0 3-0

39.925812106850145 3-0 3-1

2.2248602959962227e-10 3-0 3-2

-1.2226443908707727e-05 3-0 3-3

-7.53688222943083e-11 3-0 3-4

-2.8371507779841737e-11 3-0 3-5

2.974787083331876e-11 3-0 3-6

1.4412937640248545e-11 3-0 3-7

-1.6736612096224235e-11 3-0 3-8

-1.1256648391188986e-11 3-0 3-9

39.925812106850145 3-1 3-0

-8.710934196187736e-10 3-1 3-1

56.46361085200296 3-1 3-2

3.6836667050010874e-10 3-1 3-3

2.4452667017996286e-05 3-1 3-4

1.651789816037308e-12 3-1 3-5

6.603936147930896e-12 3-1 3-6

-6.4824812184838265e-12 3-1 3-7

-1.5431569216905604e-11 3-1 3-8

1.9558799024821383e-11 3-1 3-9

2.2248602959962227e-10 3-2 3-0

56.46361085200296 3-2 3-1

9.541842871385597e-10 3-2 3-2

-69.15350052061896 3-2 3-3

-2.4228974382367596e-10 3-2 3-4

3.8663107898805754e-05 3-2 3-5

-7.496003817664132e-11 3-2 3-6

-6.988434136934085e-11 3-2 3-7

8.300671261451953e-11 3-2 3-8

3.3946664923523447e-11 3-2 3-9

-1.2226443908485682e-05 3-3 3-0

3.6836578232168904e-10 3-3 3-1

-69.15350052061896 3-3 3-2

8.911484883356025e-10 3-3 3-3

-79.85156431620437 3-3 3-4

8.471658929920522e-10 3-3 3-5

-5.467748634555708e-05 3-3 3-6

-2.144804334136552e-10 3-3 3-7

-2.9029584291982776e-11 3-3 3-8

1.7500556559468805e-11 3-3 3-9

-7.536837820509845e-11 3-4 3-0

2.4452667016220146e-05 3-4 3-1

-2.4228974382367596e-10 3-4 3-2

-79.85156431620439 3-4 3-3

3.106400470187509e-09 3-4 3-4

89.27674063903919 3-4 3-5

7.020535264246064e-10 3-4 3-6

7.233173016910066e-05 3-4 3-7

7.421796510698186e-11 3-4 3-8

1.3145329269587273e-10 3-4 3-9

-2.8371951923261696e-11 3-5 3-0

1.6509016376176078e-12 3-5 3-1

3.866310789614122e-05 3-5 3-2

8.471587875646946e-10 3-5 3-3

89.27674063903919 3-5 3-4

-1.483208222907706e-09 3-5 3-5

97.79774496428038 3-5 3-6

3.427107486686509e-10 3-5 3-7

9.149354230331141e-05 3-5 3-8

1.6939560865125713e-10 3-5 3-9

2.974831492252861e-11 3-6 3-0

6.6056014824678335e-12 3-6 3-1

-7.49607043104561e-11 3-6 3-2

-5.467748634733344e-05 3-6 3-3

7.020553027814458e-10 3-6 3-4

97.79774496428038 3-6 3-5

-6.672564722975949e-10 3-6 3-6

105.633611254617 3-6 3-7

1.096680080081569e-09 3-6 3-8

0.00011205550193071902 3-6 3-9

1.4413048445710573e-11 3-7 3-0

-6.4824812184838265e-12 3-7 3-1

-6.988346012981505e-11 3-7 3-2

-2.144822097704946e-10 3-7 3-3

7.233173016643613e-05 3-7 3-4

3.427125250254903e-10 3-7 3-5

105.63361125461701 3-7 3-6

-3.223973976673733e-09 3-7 3-7

112.92705229254074 3-7 3-8

5.569944505623425e-10 3-7 3-9

-1.6736279029316847e-11 3-8 3-0

-1.5432453925878352e-11 3-8 3-1

8.300582443609983e-11 3-8 3-2

-2.9027752423992145e-11 3-8 3-3

7.421752101777201e-11 3-8 3-4

9.149354230597595e-05 3-8 3-5

1.0966747510110508e-09 3-8 3-6

112.92705229254074 3-8 3-7

-1.9311414689582307e-09 3-8 3-8

119.77719673373309 3-8 3-9

-1.1256624105060322e-11 3-9 3-0

1.9557244712586908e-11 3-9 3-1

3.3945096733501164e-11 3-9 3-2

1.7497447934999855e-11 3-9 3-3

1.3145529109731706e-10 3-9 3-4

1.6939472047283743e-10 3-9 3-5

0.00011205550193382764 3-9 3-6

5.569962269191819e-10 3-9 3-7

119.77719673373309 3-9 3-8

-6.792078011130798e-10 3-9 3-9

EL_ST 1 0

1.0350191814723075e-09 1-0 1-0

570.3687443835734 1-0 1-1

3.1783571330379345e-09 1-0 1-2

-0.0001746634844160739 1-0 1-3

-1.0766854074972798e-09 1-0 1-4

-4.0531911854602143e-10 1-0 1-5

4.249756102581159e-10 1-0 1-6

2.0590875879356056e-10 1-0 1-7

-2.390976305832737e-10 1-0 1-8

-1.6079823583758737e-10 1-0 1-9

570.3687443835734 1-1 1-0

-1.2444246522136382e-08 1-1 1-1

806.6230121714707 1-1 1-2

5.262293711894017e-09 1-1 1-3

0.0003493238144910392 1-1 1-4

2.3579360686198925e-11 1-1 1-5

9.436698644727001e-11 1-1 1-6

-9.262279832000786e-11 1-1 1-7

-2.204531002192251e-10 1-1 1-8

2.793845155224517e-10 1-1 1-9

3.1783571330379345e-09 1-2 1-0

806.6230121714707 1-2 1-1

1.3631279216497205e-08 1-2 1-2

-987.9071502945565 1-2 1-3

-3.4612952504176064e-09 1-2 1-4

0.0005523301128435992 1-2 1-5

-1.0708873787734774e-09 1-2 1-6

-9.983316950901027e-10 1-2 1-7

1.1858229953531918e-09 1-2 1-8

4.849214363390991e-10 1-2 1-9

-0.0001746634844160739 1-3 1-0

5.262293711894017e-09 1-3 1-1

-987.9071502945565 1-3 1-2

1.2730822618323145e-08 1-3 1-3

-1140.7366330886339 1-3 1-4

1.2102304935979191e-08 1-3 1-5

-0.0007811069477693389 1-3 1-6

-3.0639952797173464e-09 1-3 1-7

-4.1466652334095487e-10 1-3 1-8

2.4997071079724265e-10 1-3 1-9

-1.0766783020699222e-09 1-4 1-0

0.00034932381446972464 1-4 1-1

-3.461252617853461e-09 1-4 1-2

-1140.7366330886339 1-4 1-3

4.437725920070079e-08 1-4 1-4

1275.3820091291311 1-4 1-5

1.0029367558672675e-08 1-4 1-6

0.0010333104309503405 1-4 1-7

1.0602860811559367e-09 1-4 1-8

1.877965338797938e-09 1-4 1-9

-4.0531556583234263e-10 1-5 1-0

2.3579360686198925e-11 1-5 1-1

0.0005523301128436547 1-5 1-2

1.2102290725124476e-08 1-5 1-3

1275.3820091291311 1-5 1-4

-2.1188384380366188e-08 1-5 1-5

1397.1106423468623 1-5 1-6

4.8958668230625335e-09 1-5 1-7

0.001307050604340887 1-5 1-8

2.4199451331696764e-09 1-5 1-9

4.249773866149553e-10 1-6 1-0

9.4365182334855e-11 1-6 1-1

-1.0708873787734774e-09 1-6 1-2

-0.0007811069477837718 1-6 1-3

1.0029367558672675e-08 1-6 1-4

1397.1106423468623 1-6 1-5

-9.532072908768896e-09 1-6 1-6

1509.0515893516717 1-6 1-7

1.566684204590274e-08 1-6 1-8

0.0016007928846946131 1-6 1-9

2.0591052821150606e-10 1-7 1-0

-9.261214017897146e-11 1-7 1-1

-9.983315840678003e-10 1-7 1-2

-3.0639952797173464e-09 1-7 1-3

0.001033310430957446 1-7 1-4

4.8958668230625335e-09 1-7 1-5

1509.0515893516717 1-7 1-6

-4.605652748068678e-08 1-7 1-7

1613.2436041791534 1-7 1-8

7.957169145811349e-09 1-7 1-9

-2.390923015127555e-10 1-8 1-0

-2.204495475055463e-10 1-8 1-1

1.185837206207907e-09 1-8 1-2

-4.1469494505038527e-10 1-8 1-3

1.0603287137200823e-09 1-8 1-4

0.0013070506042840435 1-8 1-5

1.56668988893216e-08 1-8 1-6

```
1613.2436041791534 1-8 1-7
-2.7587361728365067e-08 1-8 1-8
1711.1028104819015 1-8 1-9
-1.608044808421009e-10 1-9 1-0
2.79383627344032e-10 1-9 1-1
4.849463053348507e-10 1-9 1-2
2.4997959258143965e-10 1-9 1-3
1.877980437831073e-09 1-9 1-4
2.4199309223149612e-09 1-9 1-5
0.0016007928847301402 1-9 1-6
7.957169145811349e-09 1-9 1-7
1711.1028104819015 1-9 1-8
-9.703057912702207e-09 1-9 1-9
9.212044460582547e-10 2-0 2-0
507.62818250138025 2-0 2-1
2.828741685334535e-09 2-0 2-2
-0.00015545050110238106 2-0 2-3
-9.582628024418227e-10 2-0 2-4
-3.6072325508562653e-10 2-0 2-5
3.7822367460194073e-10 2-0 2-6
1.8325388081996698e-10 2-0 2-7
-2.127915621485954e-10 2-0 2-8
-1.4311856560977722e-10 2-0 2-9
507.62818250138025 2-1 2-0
-1.1075371730839834e-08 2-1 2-1
717.8944808326089 2-1 2-2
4.683457177634409e-09 2-1 2-3
0.0003108981949007071 2-1 2-4
2.100009055538976e-11 2-1 2-5
8.398994000291574e-11 2-1 2-6
-8.243539184604742e-11 2-1 2-7
-1.9619911251211337e-10 2-1 2-8
2.4864910130872886e-10 2-1 2-9
2.828741685334535e-09 2-2 2-0
717.8944808326089 2-2 2-1
1.2131835092077381e-08 2-2 2-2
-879.2373637621552 2-2 2-3
-3.0805153983237687e-09 2-2 2-4
0.00049157380041423 2-2 2-5
-9.530971567528468e-10 2-2 2-6
-8.885338576014476e-10 2-2 2-7
```

1.0553691254244768e-09 2-2 2-8

4.316020874030384e-10 2-2 2-9

-0.00015545050110593378 2-3 2-0

4.683471388489124e-09 2-3 2-1

-879.2373637621552 2-3 2-2

1.133042815126828e-08 2-3 2-3

-1015.255603448884 2-3 2-4

1.0771074698823213e-08 2-3 2-5

-0.0006951851835318434 2-3 2-6

-2.7269599911505793e-09 2-3 2-7

-3.6908742728796086e-10 2-3 2-8

2.2247803599384497e-10 2-3 2-9

-9.582556970144651e-10 2-4 2-0

0.00031089819488649625 2-4 2-1

-3.0805153983237687e-09 2-4 2-2

-1015.255603448884 2-4 2-3

3.949566007577232e-08 2-4 2-4

1135.0899881249265 2-4 2-5

8.926164696276828e-09 2-4 2-6

0.0009196462835596719 2-4 2-7

9.436504910809163e-10 2-4 2-8

1.6713990191924079e-09 2-4 2-9

-3.607339123593012e-10 2-5 2-0

2.0971668845959357e-11 2-5 2-1

0.00049157380041423 2-5 2-2

1.0771060487968498e-08 2-5 2-3

1135.0899881249265 2-5 2-4

-1.8857747363654198e-08 2-5 2-5

1243.4284716887075 2-5 2-6

4.357360694484669e-09 2-5 2-7

0.0011632750378591794 2-5 2-8

2.153775824353943e-09 2-5 2-9

3.7822012188826193e-10 2-6 2-0

8.399704543027334e-11 2-6 2-1

-9.53093604039168e-10 2-6 2-2

-0.0006951851834892109 2-6 2-3

8.926164696276828e-09 2-6 2-4

1243.4284716887073 2-6 2-5

-8.483539204462431e-09 2-6 2-6

1343.0559145229872 2-6 2-7

1.3943491694590193e-08 2-6 2-8

0.001424705667353976 2-6 2-9

1.8325742832947535e-10 2-7 2-0

-8.243183913236862e-11 2-7 2-1

-8.885019386894896e-10 2-7 2-2

-2.7269528857232217e-09 2-7 2-3

0.000919646283574771 2-7 2-4

4.357389116194099e-09 2-7 2-5

1343.0559145229872 2-7 2-6

-4.099041461813613e-08 2-7 2-7

1435.7868077194462 2-7 2-8

7.081808917064336e-09 2-7 2-9

-2.127880094349166e-10 2-8 2-0

-1.9619553204285895e-10 2-8 2-1

1.0553691254244768e-09 2-8 2-2

-3.690805439052082e-10 2-8 2-3

9.436504910809163e-10 2-8 2-4

0.0011632750378804957 2-8 2-5

1.3943491694590193e-08 2-8 2-6

1435.7868077194462 2-8 2-7

-2.4552946342737414e-08 2-8 2-8

1522.8815013288922 2-8 2-9

-1.4311418022882995e-10 2-9 2-0

2.4865798309292586e-10 2-9 2-1

4.3160397478218025e-10 2-9 2-2

2.2249224684856017e-10 2-9 2-3

1.6713608275203608e-09 2-9 2-4

2.153793587922337e-09 2-9 2-5

0.0014247056673823977 2-9 2-6

7.081808917064336e-09 2-9 2-7

1522.8815013288922 2-9 2-8

-8.635652193333954e-09 2-9 2-9

-7.348575081778108e-10 3-0 3-0

-404.96180851233703 3-0 3-1

-2.25664820163729e-09 3-0 3-2

0.00012401107391750606 3-0 3-3

7.644409549811826e-10 3-0 3-4

2.8777419726691567e-10 3-0 3-5

-3.0172842002684774e-10 3-0 3-6

-1.4619140446814349e-10 3-0 3-7

1.6975132410834703e-10 3-0 3-8

1.1417012474312793e-10 3-0 3-9

-404.96180851233703 3-1 3-0

8.835399967210833e-09 3-1 3-1

-572.7023386417441 3-1 3-2

-3.736275289156765e-09 3-1 3-3

-0.0002480199083048293 3-1 3-4

-1.6758150422901963e-11 3-1 3-5

-6.698915727687579e-11 3-1 3-6

6.575984201617757e-11 3-1 3-7

1.5650689078050132e-10 3-1 3-8

-1.9837287368318357e-10 3-1 3-9

-2.25664820163729e-09 3-2 3-0

-572.7023386417441 3-2 3-1

-9.678302603788325e-09 3-2 3-2

701.4140767091351 3-2 3-3

2.457511527609313e-09 3-2 3-4

-0.00039215438014389425 3-2 3-5

7.603269125411316e-10 3-2 3-6

7.088316600345388e-10 3-2 3-7

-8.419238639589821e-10 3-2 3-8

-3.443039187091035e-10 3-2 3-9

0.0001240110739281642 3-3 3-0

-3.73626107830205e-09 3-3 3-1

701.4140767091351 3-3 3-2

-9.038927828441956e-09 3-3 3-3

809.9230094929299 3-3 3-4

-8.592593303546892e-09 3-3 3-5

0.0005545859329348923 3-3 3-6

2.175461588649341e-09 3-3 3-7

2.944691157582455e-10 3-3 3-8

-1.7749535174971243e-10 3-3 3-9

7.644480604085402e-10 3-4 3-0

-0.0002480199083048293 3-4 3-1

2.457511527609313e-09 3-4 3-2

809.9230094929299 3-4 3-3

-3.1507795483776135e-08 3-4 3-4

-905.521226481683 3-4 3-5

-7.120902978385857e-09 3-4 3-6

-0.0007336504059813898 3-4 3-7

-7.528342393925413e-10 3-4 3-8

-1.3333485426909203e-09 3-4 3-9

2.8777774911323273e-10 3-5 3-0

-1.6722623286113958e-11 3-5 3-1

-0.000392154380143922 3-5 3-2

-8.592621725256322e-09 3-5 3-3

-905.521226481683 3-5 3-4

1.5043895018607145e-08 3-5 3-5

-991.9485560662722 3-5 3-6

-3.4760603284667013e-09 3-5 3-7

-0.0009280059291025822 3-5 3-8

-1.7181776001962135e-09 3-5 3-9

-3.0173197274052654e-10 3-6 3-0

-6.699094057260909e-11 3-6 3-1

7.603127016864164e-10 3-6 3-2

0.0005545859329350034 3-6 3-3

-7.120902978385857e-09 3-6 3-4

-991.9485560662722 3-6 3-5

6.767834292986663e-09 3-6 3-6

-1071.4266284396865 3-6 3-7

-1.1123418630631932e-08 3-6 3-8

-0.0011365629481794315 3-6 3-9

-1.4618607713234876e-10 3-7 3-0

6.577050015721397e-11 3-7 3-1

7.088210574046536e-10 3-7 3-2

2.1754509305083047e-09 3-7 3-3

-0.000733650406002706 3-7 3-4

-3.4760745393214165e-09 3-7 3-5

-1071.4266284396865 3-7 3-6

3.270037041147589e-08 3-7 3-7

-1145.4029589671986 3-7 3-8

-5.649610557156848e-09 3-7 3-9

1.6975398864360614e-10 3-8 3-0

1.5652465434889533e-10 3-8 3-1

-8.419238639589821e-10 3-8 3-2

2.9449065408492323e-10 3-8 3-3

-7.528129231104685e-10 3-8 3-4

-0.0009280059290439624 3-8 3-5

-1.1123390208922501e-08 3-8 3-6

-1145.4029589671986 3-8 3-7

1.9587162114476087e-08 3-8 3-8

-1214.8829954421499 3-8 3-9

1.1417371215127625e-10 3-9 3-0

-1.9837198550476387e-10 3-9 3-1

-3.4430713835587493e-10 3-9 3-2

-1.7749179903603363e-10 3-9 3-3

-1.3333423254419824e-09 3-9 3-4

-1.7181882583372499e-09 3-9 3-5

-0.0011365629481403516 3-9 3-6

-5.649638978866278e-09 3-9 3-7

-1214.8829954421499 3-9 3-8

6.889194992254488e-09 3-9 3-9

-80.67720500110065 4-0 4-0

-274.6680287967075 4-0 4-1

-12.010391278256833 4-0 4-2

0.8593235436280906 4-0 4-3

-0.08002964444018268 4-0 4-4

0.008906472534873533 4-0 4-5

-0.0011333400832009843 4-0 4-6

0.000160548399862126 4-0 4-7

-2.4870668870136098e-05 4-0 4-8

4.159692352789863e-06 4-0 4-9

-274.6680287967075 4-1 4-0

-29.5571831917197 4-1 4-1

-389.1811548086439 4-1 4-2

20.882274167337364 4-1 4-3

-1.7285437948777904 4-1 4-4

0.1803290111095226 4-1 4-5

-0.022026702131555886 4-1 4-6

0.0030333192671032627 4-1 4-7

-0.00046025925455106564 4-1 4-8

7.577155095739188e-05 4-1 4-9

-12.010391278256833 4-2 4-0

-389.1811548086439 4-2 4-1

22.022637565509626 4-2 4-2

477.5614952532045 4-2 4-3

-29.64552768069303 4-2 4-4

2.7488667750644176 4-2 4-5

-0.3147522329768204 4-2 4-6

0.04160713172237429 4-2 4-7

-0.0061372883604859894 4-2 4-8

0.0009896556415576318 4-2 4-9

0.859323543628087 4-3 4-0

20.882274167337364 4-3 4-1

477.5614952532045 4-3 4-2

74.06991035835148 4-3 4-3

552.5018606339215 4-3 4-4

-38.41991382913489 4-3 4-5

3.910044342312771 4-3 4-6

-0.4845212034399893 4-3 4-7

0.06860452175037149 4-3 4-8

-0.01075437523091804 4-3 4-9

-0.08002964444018268 4-4 4-0

-1.7285437948777833 4-4 4-1

-29.64552768069302 4-4 4-2

552.5018606339215 4-4 4-3

126.59247388269938 4-4 4-4

618.909360805626 4-4 4-5

-47.236893093841786 4-4 4-6

5.20263631074986 4-4 4-7

-0.6905520820713524 4-4 4-8

0.10391087649592108 4-4 4-9

0.008906472534873089 4-5 4-0

0.18032901110951904 4-5 4-1

2.7488667750644176 4-5 4-2

-38.4199138291349 4-5 4-3

618.909360805626 4-5 4-4

179.59835852725752 4-5 4-5

679.2962635261858 4-5 4-6

-56.108826327325325 4-5 4-7

6.6193609305617205 4-5 4-8

-0.933715394806252 4-5 4-9

-0.001133340083204537 4-6 4-0

-0.022026702131552334 4-6 4-1

-0.31475223297681687 4-6 4-2

3.9100443423127746 4-6 4-3

-47.2368930938418 4-6 4-4

679.2962635261858 4-6 4-5

233.0957923378919 4-6 4-6

735.152517484506 4-6 4-7

-65.04186361861703 4-6 4-8

8.154629116275354 4-6 4-9

0.00016054839985946145 4-7 4-0

0.00303331926710948 4-7 4-1

0.04160713172238495 4-7 4-2

-0.48452120343998395 4-7 4-3

5.202636310749856 4-7 4-4

-56.108826327325325 4-7 4-5

735.152517484506 4-7 4-6

287.0932073861537 4-7 4-7

787.4473988776481 4-7 4-8

-74.0396325085147 4-7 4-9

-2.4870668871912454e-05 4-8 4-0

-0.00046025925455017746 4-8 4-1

-0.0061372883604815485 4-8 4-2

0.0686045217503484 4-8 4-3

-0.6905520820713578 4-8 4-4

6.619360930561745 4-8 4-5

-65.04186361861703 4-8 4-6

787.4473988776481 4-8 4-7

341.59923926910494 4-8 4-8

836.8531818649661 4-8 4-9

4.159692355454398e-06 4-9 4-0

7.577155095916824e-05 4-9 4-1

0.000989655641554079 4-9 4-2

-0.010754375230910935 4-9 4-3

0.10391087649592819 4-9 4-4

-0.9337153948062449 4-9 4-5

8.154629116275318 4-9 4-6

-74.0396325085147 4-9 4-7

836.853181864966 4-9 4-8

396.62263529758013 4-9 4-9

89.93048279628223 5-0 5-0

160.38081572289633 5-0 5-1

-2.577800777394227 5-0 5-2

0.0677257183148976 5-0 5-3

-0.002313852610271727 5-0 5-4

9.437840579806789e-05 5-0 5-5

-4.39746654132267e-06 5-0 5-6

2.278029023194971e-07 5-0 5-7

-1.2867856558070437e-08 5-0 5-8

8.465905754333616e-10 5-0 5-9

160.38081572289633 5-1 5-0

100.87189610340774 5-1 5-1

226.87125653063833 5-1 5-2

-4.467182467541896 5-1 5-3

0.135555856671985 5-1 5-4

-0.0051792344178185346 5-1 5-5

0.00023147378813925812 5-1 5-6

-1.1652279591212166e-05 5-1 5-7

6.457967325346649e-07 5-1 5-8

-3.8897015719313676e-08 5-1 5-9

-2.577800777394227 5-2 5-0

226.87125653063833 5-2 5-1

111.82650383711918 5-2 5-2

277.93115025776297 5-2 5-3

-6.320806885983895 5-2 5-4

0.2144979441345809 5-2 5-5

-0.008979895821394912 5-2 5-6

0.00043360179985008085 5-2 5-7

-2.334060974518123e-05 5-2 5-8

1.3720165701348692e-06 5-2 5-9

0.06772571831490115 5-3 5-0

-4.4671824675418925 5-3 5-1

277.93115025776297 5-3 5-2

122.79433528554297 5-3 5-3

321.0101543446059 5-3 5-4

-8.164335560897786 5-3 5-5

0.30358000127820617 5-3 5-6

-0.013731092073959061 5-3 5-7

0.0007089733761587193 5-3 5-8

-4.0488747055969714e-05 5-3 5-9

-0.002313852610271727 5-4 5-0

0.13555585667198589 5-4 5-1

-6.320806885983888 5-4 5-2

321.0101543446059 5-4 5-3

133.77541982851733 5-4 5-4

358.99302674669775 5-4 5-5

-10.00438838763305 5-4 5-6

0.4019086772298781 5-4 5-7

-0.01943863126750943 5-4 5-8

0.0010648203194581383 5-4 5-9

9.437840579806789e-05 5-5 5-0

-0.005179234417820311 5-5 5-1

0.21449794413457823 5-5 5-2

-8.164335560897783 5-5 5-3

358.99302674669775 5-5 5-4

144.76978696973822 5-5 5-5

393.3588540397019 5-5 5-6

-11.843464071049091 5-5 5-7

0.5087714734517857 5-5 5-8

-0.02610644738115364 5-5 5-9

-4.397466538630379e-06 5-6 5-0

0.0002314737881401463 5-6 5-1

-0.008979895821403794 5-6 5-2

0.3035800012782097 5-6 5-3

-10.00438838763305 5-6 5-4

393.3588540397019 5-6 5-5

155.7774662741857 5-6 5-6

424.98605300704855 5-6 5-7

-13.682719341271309 5-6 5-8

0.6235968964734734 5-6 5-9

2.2780290542812157e-07 5-7 5-0

-1.1652279591212166e-05 5-7 5-1

0.0004336017998518572 5-7 5-2

-0.013731092073955509 5-7 5-3

0.4019086772298781 5-7 5-4

-11.843464071049112 5-7 5-5

424.98605300704855 5-7 5-6

166.79848741240167 5-7 5-7

454.4468138118923 5-7 5-8

-15.522764701306432 5-7 5-9

-1.2867854337624388e-08 5-8 5-0

6.457967325346649e-07 5-8 5-1

-2.3340609734967178e-05 5-8 5-2

0.000708973376162272 5-8 5-3

-0.019438631267507653 5-8 5-4

0.5087714734517893 5-8 5-5

-13.682719341271302 5-8 5-6

454.4468138118923 5-8 5-7

177.83288004456205 5-8 5-8

482.1384799253084 5-8 5-9

8.465919077009912e-10 5-9 5-0

-3.8897014775624105e-08 5-9 5-1

1.3720165772402965e-06 5-9 5-2

-4.0488747061298784e-05 5-9 5-3

0.0010648203194625792 5-9 5-4

-0.026106447381160747 5-9 5-5

0.6235968964734734 5-9 5-6

-15.52276470130644 5-9 5-7

482.1384799253084 5-9 5-8

188.88067260506608 5-9 5-9

41.158674803481624 6-0 6-0

166.9028837596028 6-0 6-1

-2.8640250882142624 6-0 6-2

0.0803289134152152 6-0 6-3

-0.0029296463038361864 6-0 6-4

0.00012755028233213395 6-0 6-5

-6.343126138119715e-06 6-0 6-6

3.507595103258156e-07 6-0 6-7

-2.1246453152378692e-08 6-0 6-8

1.4507310908149777e-09 6-0 6-9

166.9028837596028 6-1 6-0

53.31569391722762 6-1 6-1

236.10575738892214 6-1 6-2

-4.963551573602373 6-1 6-3

0.1607991172255785 6-1 6-4

-0.006558550881877068 6-1 6-5

0.0003128885614382959 6-1 6-6

-1.6811951609518868e-05 6-1 6-7

9.944116947657805e-07 6-1 6-8

-6.372066851942651e-08 6-1 6-9

-2.864025088214266 6-2 6-0

236.10575738892214 6-2 6-1

65.4894273637104 6-2 6-2

289.2544318047409 6-2 6-3

-7.02364871315266 6-2 6-4

0.25446945831484324 6-2 6-5

-0.01137304052363941 6-2 6-6

0.0005862157496450138 6-2 6-7

-3.368244399482017e-05 6-2 6-8

2.113335732190292e-06 6-2 6-9

0.08032891341521697 6-3 6-0

-4.96355157360237 6-3 6-1

289.2544318047409 6-3 6-2

77.67991743137193 6-3 6-3

334.1006192884814 6-3 6-4

-9.072825450457955 6-3 6-5

0.360191033843706 6-3 6-6

-0.0173929530158059 6-3 6-7

0.000958683573508257 6-3 6-8

-5.844190811243732e-05 6-3 6-9

-0.0029296463038361864 6-4 6-0

0.1607991172255785 6-4 6-1

-7.02364871315266 6-4 6-2

334.1006192884814 6-4 6-3

89.88720660115014 6-4 6-4

373.64592803547606 6-4 6-5

-11.118436921248858 6-4 6-6

0.47690775477216274 6-4 6-7

-0.024626177194360107 6-4 6-8

0.0014401268854102951 6-4 6-9

0.00012755028233257804 6-5 6-0

-0.006558550881877068 6-5 6-1

0.2544694583148468 6-5 6-2

-9.07282545045797 6-5 6-3

373.64592803547606 6-5 6-4

102.11133746348824 6-5 6-5

409.42930042601745 6-5 6-6

-13.163259981459504 6-5 6-7

0.6037777027957851 6-5 6-8

-0.03307822996863226 6-5 6-9

-6.343126137675625e-06 6-6 6-0

0.00031288856143651955 6-6 6-1

-0.011373040523645628 6-6 6-2

0.36019103384370954 6-6 6-3

-11.118436921248858 6-6 6-4

409.42930042601745 6-6 6-5

114.35235282121945 6-6 6-6

442.36466857992434 6-6 6-7

-15.208580658635768 6-6 6-8

0.7401259447701689 6-6 6-9

3.507595103258156e-07 6-7 6-0

-1.681195161440385e-05 6-7 6-1

0.0005862157496476783 6-7 6-2

-0.017392953015800572 6-7 6-3

0.4769077547721574 6-7 6-4

-13.163259981459532 6-7 6-5

442.36466857992434 6-7 6-6

126.61029562957819 6-7 6-7

473.0473361389812 6-7 6-8

-17.25507817212126 6-7 6-9

-2.1246454262601717e-08 6-8 6-0

9.944116947657805e-07 6-8 6-1

-3.3682443993043815e-05 6-8 6-2

0.0009586835735033721 6-8 6-3

-0.024626177194370765 6-8 6-4

0.6037777027957869 6-8 6-5

-15.208580658635775 6-8 6-6

473.0473361389812 6-8 6-7

138.88520888562502 6-8 6-8

501.89068297028183 6-8 6-9

1.450730202636558e-09 6-9 6-0

-6.372066763124809e-08 6-9 6-1

2.1133357268612215e-06 6-9 6-2

-5.844190811643412e-05 6-9 6-3

0.0014401268854085188 6-9 6-4

-0.03307822996863226 6-9 6-5

0.740125944770176 6-9 6-6

-17.25507817212126 6-9 6-7

501.89068297028183 6-9 6-8

151.17713556953166 6-9 6-9

EL_ST 0 1

1.0350191814723075e-09 1-0 1-0

570.3687443835734 1-0 1-1

3.1783571330379345e-09 1-0 1-2

-0.0001746634844160739 1-0 1-3

-1.0766854074972798e-09 1-0 1-4

-4.0531911854602143e-10 1-0 1-5

4.249756102581159e-10 1-0 1-6

2.0590875879356056e-10 1-0 1-7

-2.390976305832737e-10 1-0 1-8

-1.6079823583758737e-10 1-0 1-9

570.3687443835734 1-1 1-0

-1.2444246522136382e-08 1-1 1-1

806.6230121714707 1-1 1-2

5.262293711894017e-09 1-1 1-3

0.0003493238144910392 1-1 1-4

2.3579360686198925e-11 1-1 1-5

9.436698644727001e-11 1-1 1-6

-9.262279832000786e-11 1-1 1-7

-2.204531002192251e-10 1-1 1-8

2.793845155224517e-10 1-1 1-9

3.1783571330379345e-09 1-2 1-0

806.6230121714707 1-2 1-1

1.3631279216497205e-08 1-2 1-2

-987.9071502945565 1-2 1-3

-3.4612952504176064e-09 1-2 1-4

0.0005523301128435992 1-2 1-5

-1.0708873787734774e-09 1-2 1-6

-9.983316950901027e-10 1-2 1-7

1.1858229953531918e-09 1-2 1-8

4.849214363390991e-10 1-2 1-9

-0.0001746634844160739 1-3 1-0

5.262293711894017e-09 1-3 1-1

-987.9071502945565 1-3 1-2

1.2730822618323145e-08 1-3 1-3

-1140.7366330886339 1-3 1-4

1.2102304935979191e-08 1-3 1-5

-0.0007811069477693389 1-3 1-6

-3.0639952797173464e-09 1-3 1-7

-4.1466652334095487e-10 1-3 1-8

2.4997071079724265e-10 1-3 1-9

-1.0766783020699222e-09 1-4 1-0

0.00034932381446972464 1-4 1-1

-3.461252617853461e-09 1-4 1-2

-1140.7366330886339 1-4 1-3

4.437725920070079e-08 1-4 1-4

1275.3820091291311 1-4 1-5

1.0029367558672675e-08 1-4 1-6

0.0010333104309503405 1-4 1-7

1.0602860811559367e-09 1-4 1-8

1.877965338797938e-09 1-4 1-9

-4.0531556583234263e-10 1-5 1-0

2.3579360686198925e-11 1-5 1-1

0.0005523301128436547 1-5 1-2

1.2102290725124476e-08 1-5 1-3

1275.3820091291311 1-5 1-4

-2.1188384380366188e-08 1-5 1-5

1397.1106423468623 1-5 1-6

4.8958668230625335e-09 1-5 1-7

0.001307050604340887 1-5 1-8

2.4199451331696764e-09 1-5 1-9

4.249773866149553e-10 1-6 1-0

9.4365182334855e-11 1-6 1-1

-1.0708873787734774e-09 1-6 1-2

-0.0007811069477837718 1-6 1-3

1.0029367558672675e-08 1-6 1-4

1397.1106423468623 1-6 1-5

-9.532072908768896e-09 1-6 1-6

1509.0515893516717 1-6 1-7

1.566684204590274e-08 1-6 1-8

0.0016007928846946131 1-6 1-9

2.0591052821150606e-10 1-7 1-0

-9.261214017897146e-11 1-7 1-1

-9.983315840678003e-10 1-7 1-2

-3.0639952797173464e-09 1-7 1-3

0.001033310430957446 1-7 1-4

4.8958668230625335e-09 1-7 1-5

1509.0515893516717 1-7 1-6

-4.605652748068678e-08 1-7 1-7

1613.2436041791534 1-7 1-8

7.957169145811349e-09 1-7 1-9

-2.390923015127555e-10 1-8 1-0

-2.204495475055463e-10 1-8 1-1

1.185837206207907e-09 1-8 1-2

-4.1469494505038527e-10 1-8 1-3

1.0603287137200823e-09 1-8 1-4

0.0013070506042840435 1-8 1-5

1.56668988893216e-08 1-8 1-6

1613.2436041791534 1-8 1-7

-2.7587361728365067e-08 1-8 1-8

1711.1028104819015 1-8 1-9

-1.608044808421009e-10 1-9 1-0

2.79383627344032e-10 1-9 1-1

4.849463053348507e-10 1-9 1-2

2.4997959258143965e-10 1-9 1-3

1.877980437831073e-09 1-9 1-4

2.4199309223149612e-09 1-9 1-5

0.0016007928847301402 1-9 1-6

7.957169145811349e-09 1-9 1-7

1711.1028104819015 1-9 1-8

-9.703057912702207e-09 1-9 1-9

9.212044460582547e-10 2-0 2-0

507.62818250138025 2-0 2-1

2.828741685334535e-09 2-0 2-2

-0.00015545050110238106 2-0 2-3

-9.582628024418227e-10 2-0 2-4

-3.6072325508562653e-10 2-0 2-5

3.7822367460194073e-10 2-0 2-6

1.8325388081996698e-10 2-0 2-7

-2.127915621485954e-10 2-0 2-8

-1.4311856560977722e-10 2-0 2-9

507.62818250138025 2-1 2-0

-1.1075371730839834e-08 2-1 2-1

717.8944808326089 2-1 2-2

4.683457177634409e-09 2-1 2-3

0.0003108981949007071 2-1 2-4

2.100009055538976e-11 2-1 2-5

8.398994000291574e-11 2-1 2-6

-8.243539184604742e-11 2-1 2-7

-1.9619911251211337e-10 2-1 2-8

2.4864910130872886e-10 2-1 2-9

2.828741685334535e-09 2-2 2-0

717.8944808326089 2-2 2-1

1.2131835092077381e-08 2-2 2-2

-879.2373637621552 2-2 2-3

-3.0805153983237687e-09 2-2 2-4

0.00049157380041423 2-2 2-5

-9.530971567528468e-10 2-2 2-6

-8.885338576014476e-10 2-2 2-7

1.0553691254244768e-09 2-2 2-8

4.316020874030384e-10 2-2 2-9

-0.00015545050110593378 2-3 2-0

4.683471388489124e-09 2-3 2-1

-879.2373637621552 2-3 2-2

1.133042815126828e-08 2-3 2-3

-1015.255603448884 2-3 2-4

1.0771074698823213e-08 2-3 2-5

-0.0006951851835318434 2-3 2-6

-2.7269599911505793e-09 2-3 2-7

-3.6908742728796086e-10 2-3 2-8

2.2247803599384497e-10 2-3 2-9

-9.582556970144651e-10 2-4 2-0

0.00031089819488649625 2-4 2-1

-3.0805153983237687e-09 2-4 2-2

-1015.255603448884 2-4 2-3

3.949566007577232e-08 2-4 2-4

1135.0899881249265 2-4 2-5

8.926164696276828e-09 2-4 2-6

0.0009196462835596719 2-4 2-7

9.436504910809163e-10 2-4 2-8

1.6713990191924079e-09 2-4 2-9

-3.607339123593012e-10 2-5 2-0

2.0971668845959357e-11 2-5 2-1

0.00049157380041423 2-5 2-2

1.0771060487968498e-08 2-5 2-3

1135.0899881249265 2-5 2-4

-1.8857747363654198e-08 2-5 2-5

1243.4284716887075 2-5 2-6

4.357360694484669e-09 2-5 2-7

0.0011632750378591794 2-5 2-8

2.153775824353943e-09 2-5 2-9

3.7822012188826193e-10 2-6 2-0

8.399704543027334e-11 2-6 2-1

-9.53093604039168e-10 2-6 2-2

-0.0006951851834892109 2-6 2-3

8.926164696276828e-09 2-6 2-4

1243.4284716887073 2-6 2-5

-8.483539204462431e-09 2-6 2-6

1343.0559145229872 2-6 2-7

1.3943491694590193e-08 2-6 2-8

0.001424705667353976 2-6 2-9

1.8325742832947535e-10 2-7 2-0

-8.243183913236862e-11 2-7 2-1

-8.885019386894896e-10 2-7 2-2

-2.7269528857232217e-09 2-7 2-3

0.000919646283574771 2-7 2-4

4.357389116194099e-09 2-7 2-5

1343.0559145229872 2-7 2-6

-4.099041461813613e-08 2-7 2-7

1435.7868077194462 2-7 2-8

7.081808917064336e-09 2-7 2-9

-2.127880094349166e-10 2-8 2-0

-1.9619553204285895e-10 2-8 2-1

1.0553691254244768e-09 2-8 2-2

-3.690805439052082e-10 2-8 2-3

9.436504910809163e-10 2-8 2-4

0.0011632750378804957 2-8 2-5

1.3943491694590193e-08 2-8 2-6

1435.7868077194462 2-8 2-7

-2.4552946342737414e-08 2-8 2-8

1522.8815013288922 2-8 2-9

-1.4311418022882995e-10 2-9 2-0

2.4865798309292586e-10 2-9 2-1

4.3160397478218025e-10 2-9 2-2

2.2249224684856017e-10 2-9 2-3

1.6713608275203608e-09 2-9 2-4

2.153793587922337e-09 2-9 2-5

0.0014247056673823977 2-9 2-6

7.081808917064336e-09 2-9 2-7

1522.8815013288922 2-9 2-8

-8.635652193333954e-09 2-9 2-9

-7.348575081778108e-10 3-0 3-0

-404.96180851233703 3-0 3-1

-2.25664820163729e-09 3-0 3-2

0.00012401107391750606 3-0 3-3

7.644409549811826e-10 3-0 3-4

2.8777419726691567e-10 3-0 3-5

-3.0172842002684774e-10 3-0 3-6

-1.4619140446814349e-10 3-0 3-7

1.6975132410834703e-10 3-0 3-8

1.1417012474312793e-10 3-0 3-9

-404.96180851233703 3-1 3-0

8.835399967210833e-09 3-1 3-1

-572.7023386417441 3-1 3-2

-3.736275289156765e-09 3-1 3-3

-0.0002480199083048293 3-1 3-4

-1.6758150422901963e-11 3-1 3-5

-6.698915727687579e-11 3-1 3-6

6.575984201617757e-11 3-1 3-7

1.5650689078050132e-10 3-1 3-8

-1.9837287368318357e-10 3-1 3-9

-2.25664820163729e-09 3-2 3-0

-572.7023386417441 3-2 3-1

-9.678302603788325e-09 3-2 3-2

701.4140767091351 3-2 3-3

2.457511527609313e-09 3-2 3-4

-0.00039215438014389425 3-2 3-5

7.603269125411316e-10 3-2 3-6

7.088316600345388e-10 3-2 3-7

-8.419238639589821e-10 3-2 3-8

-3.443039187091035e-10 3-2 3-9

0.0001240110739281642 3-3 3-0

-3.73626107830205e-09 3-3 3-1

701.4140767091351 3-3 3-2

-9.038927828441956e-09 3-3 3-3

809.9230094929299 3-3 3-4

-8.592593303546892e-09 3-3 3-5

0.0005545859329348923 3-3 3-6

2.175461588649341e-09 3-3 3-7

2.944691157582455e-10 3-3 3-8

-1.7749535174971243e-10 3-3 3-9

7.644480604085402e-10 3-4 3-0

-0.0002480199083048293 3-4 3-1

2.457511527609313e-09 3-4 3-2

809.9230094929299 3-4 3-3

-3.1507795483776135e-08 3-4 3-4

-905.521226481683 3-4 3-5

-7.120902978385857e-09 3-4 3-6

-0.0007336504059813898 3-4 3-7

-7.528342393925413e-10 3-4 3-8

-1.3333485426909203e-09 3-4 3-9

2.8777774911323273e-10 3-5 3-0

-1.6722623286113958e-11 3-5 3-1

-0.000392154380143922 3-5 3-2

-8.592621725256322e-09 3-5 3-3

-905.521226481683 3-5 3-4

1.5043895018607145e-08 3-5 3-5

-991.9485560662722 3-5 3-6

-3.4760603284667013e-09 3-5 3-7

-0.0009280059291025822 3-5 3-8

-1.7181776001962135e-09 3-5 3-9

-3.0173197274052654e-10 3-6 3-0

-6.699094057260909e-11 3-6 3-1

7.603127016864164e-10 3-6 3-2

0.0005545859329350034 3-6 3-3

-7.120902978385857e-09 3-6 3-4

-991.9485560662722 3-6 3-5

6.767834292986663e-09 3-6 3-6

-1071.4266284396865 3-6 3-7

-1.1123418630631932e-08 3-6 3-8

-0.0011365629481794315 3-6 3-9

-1.4618607713234876e-10 3-7 3-0

6.577050015721397e-11 3-7 3-1

7.088210574046536e-10 3-7 3-2

2.1754509305083047e-09 3-7 3-3

-0.000733650406002706 3-7 3-4

-3.4760745393214165e-09 3-7 3-5

-1071.4266284396865 3-7 3-6

3.270037041147589e-08 3-7 3-7

-1145.4029589671986 3-7 3-8

-5.649610557156848e-09 3-7 3-9

1.6975398864360614e-10 3-8 3-0

1.5652465434889533e-10 3-8 3-1

-8.419238639589821e-10 3-8 3-2

2.9449065408492323e-10 3-8 3-3

-7.528129231104685e-10 3-8 3-4

-0.0009280059290439624 3-8 3-5

-1.1123390208922501e-08 3-8 3-6

-1145.4029589671986 3-8 3-7

1.9587162114476087e-08 3-8 3-8

-1214.8829954421499 3-8 3-9

1.1417371215127625e-10 3-9 3-0

-1.9837198550476387e-10 3-9 3-1

-3.4430713835587493e-10 3-9 3-2

-1.7749179903603363e-10 3-9 3-3

-1.3333423254419824e-09 3-9 3-4

-1.7181882583372499e-09 3-9 3-5

-0.0011365629481403516 3-9 3-6

-5.649638978866278e-09 3-9 3-7

-1214.8829954421499 3-9 3-8

6.889194992254488e-09 3-9 3-9

-80.67720500110065 4-0 4-0

-274.6680287967075 4-0 4-1

-12.010391278256833 4-0 4-2

0.8593235436280906 4-0 4-3

-0.08002964444018268 4-0 4-4

0.008906472534873533 4-0 4-5

-0.0011333400832009843 4-0 4-6

0.000160548399862126 4-0 4-7

-2.4870668870136098e-05 4-0 4-8

4.159692352789863e-06 4-0 4-9

-274.6680287967075 4-1 4-0

-29.5571831917197 4-1 4-1

-389.1811548086439 4-1 4-2

20.882274167337364 4-1 4-3

-1.7285437948777904 4-1 4-4

0.1803290111095226 4-1 4-5

-0.022026702131555886 4-1 4-6

0.0030333192671032627 4-1 4-7

-0.00046025925455106564 4-1 4-8

7.577155095739188e-05 4-1 4-9

-12.010391278256833 4-2 4-0

-389.1811548086439 4-2 4-1

22.022637565509626 4-2 4-2

477.5614952532045 4-2 4-3

-29.64552768069303 4-2 4-4

2.7488667750644176 4-2 4-5

-0.3147522329768204 4-2 4-6

0.04160713172237429 4-2 4-7

-0.0061372883604859894 4-2 4-8

0.0009896556415576318 4-2 4-9

0.859323543628087 4-3 4-0

20.882274167337364 4-3 4-1

477.5614952532045 4-3 4-2

74.06991035835148 4-3 4-3

552.5018606339215 4-3 4-4

-38.41991382913489 4-3 4-5

3.910044342312771 4-3 4-6

-0.4845212034399893 4-3 4-7

0.06860452175037149 4-3 4-8

-0.01075437523091804 4-3 4-9

-0.08002964444018268 4-4 4-0

-1.7285437948777833 4-4 4-1

-29.64552768069302 4-4 4-2

552.5018606339215 4-4 4-3

126.59247388269938 4-4 4-4

618.909360805626 4-4 4-5

-47.236893093841786 4-4 4-6

5.20263631074986 4-4 4-7

-0.6905520820713524 4-4 4-8

0.10391087649592108 4-4 4-9

0.008906472534873089 4-5 4-0

0.18032901110951904 4-5 4-1

2.7488667750644176 4-5 4-2

-38.4199138291349 4-5 4-3

618.909360805626 4-5 4-4

179.59835852725752 4-5 4-5

679.2962635261858 4-5 4-6

-56.108826327325325 4-5 4-7

6.6193609305617205 4-5 4-8

-0.933715394806252 4-5 4-9

-0.001133340083204537 4-6 4-0

-0.022026702131552334 4-6 4-1

-0.31475223297681687 4-6 4-2

3.9100443423127746 4-6 4-3

-47.2368930938418 4-6 4-4

679.2962635261858 4-6 4-5

233.0957923378919 4-6 4-6

735.152517484506 4-6 4-7

-65.04186361861703 4-6 4-8

8.154629116275354 4-6 4-9

0.00016054839985946145 4-7 4-0

0.00303331926710948 4-7 4-1

0.04160713172238495 4-7 4-2

-0.48452120343998395 4-7 4-3

5.202636310749856 4-7 4-4

-56.108826327325325 4-7 4-5

735.152517484506 4-7 4-6

287.0932073861537 4-7 4-7

787.4473988776481 4-7 4-8

-74.0396325085147 4-7 4-9

-2.4870668871912454e-05 4-8 4-0

-0.00046025925455017746 4-8 4-1

-0.0061372883604815485 4-8 4-2

0.0686045217503484 4-8 4-3

-0.6905520820713578 4-8 4-4

6.619360930561745 4-8 4-5

-65.04186361861703 4-8 4-6

787.4473988776481 4-8 4-7

341.59923926910494 4-8 4-8

836.8531818649661 4-8 4-9

4.159692355454398e-06 4-9 4-0

7.577155095916824e-05 4-9 4-1

0.000989655641554079 4-9 4-2

-0.010754375230910935 4-9 4-3

0.10391087649592819 4-9 4-4

-0.9337153948062449 4-9 4-5

8.154629116275318 4-9 4-6

-74.0396325085147 4-9 4-7

836.853181864966 4-9 4-8

396.62263529758013 4-9 4-9

89.93048279628223 5-0 5-0

160.38081572289633 5-0 5-1

-2.577800777394227 5-0 5-2

0.0677257183148976 5-0 5-3

-0.002313852610271727 5-0 5-4

9.437840579806789e-05 5-0 5-5

-4.39746654132267e-06 5-0 5-6

2.278029023194971e-07 5-0 5-7

-1.2867856558070437e-08 5-0 5-8

8.465905754333616e-10 5-0 5-9

160.38081572289633 5-1 5-0

100.87189610340774 5-1 5-1

226.87125653063833 5-1 5-2

-4.467182467541896 5-1 5-3

0.135555856671985 5-1 5-4

-0.0051792344178185346 5-1 5-5

0.00023147378813925812 5-1 5-6

-1.1652279591212166e-05 5-1 5-7

6.457967325346649e-07 5-1 5-8

-3.8897015719313676e-08 5-1 5-9

-2.577800777394227 5-2 5-0

226.87125653063833 5-2 5-1

111.82650383711918 5-2 5-2

277.93115025776297 5-2 5-3

-6.320806885983895 5-2 5-4

0.2144979441345809 5-2 5-5

-0.008979895821394912 5-2 5-6

0.00043360179985008085 5-2 5-7

-2.334060974518123e-05 5-2 5-8

1.3720165701348692e-06 5-2 5-9

0.06772571831490115 5-3 5-0

-4.4671824675418925 5-3 5-1

277.93115025776297 5-3 5-2

122.79433528554297 5-3 5-3

321.0101543446059 5-3 5-4

-8.164335560897786 5-3 5-5

0.30358000127820617 5-3 5-6

-0.013731092073959061 5-3 5-7

0.0007089733761587193 5-3 5-8

-4.0488747055969714e-05 5-3 5-9

-0.002313852610271727 5-4 5-0

0.13555585667198589 5-4 5-1

-6.320806885983888 5-4 5-2

321.0101543446059 5-4 5-3

133.77541982851733 5-4 5-4

358.99302674669775 5-4 5-5

-10.00438838763305 5-4 5-6

0.4019086772298781 5-4 5-7

-0.01943863126750943 5-4 5-8

0.0010648203194581383 5-4 5-9

9.437840579806789e-05 5-5 5-0

-0.005179234417820311 5-5 5-1

0.21449794413457823 5-5 5-2

-8.164335560897783 5-5 5-3

358.99302674669775 5-5 5-4

144.76978696973822 5-5 5-5

393.3588540397019 5-5 5-6

-11.843464071049091 5-5 5-7

0.5087714734517857 5-5 5-8

-0.02610644738115364 5-5 5-9

-4.397466538630379e-06 5-6 5-0

0.0002314737881401463 5-6 5-1

-0.008979895821403794 5-6 5-2

0.3035800012782097 5-6 5-3

-10.00438838763305 5-6 5-4

393.3588540397019 5-6 5-5

155.7774662741857 5-6 5-6

424.98605300704855 5-6 5-7

-13.682719341271309 5-6 5-8

0.6235968964734734 5-6 5-9

2.2780290542812157e-07 5-7 5-0

-1.1652279591212166e-05 5-7 5-1

0.0004336017998518572 5-7 5-2

-0.013731092073955509 5-7 5-3

0.4019086772298781 5-7 5-4

-11.843464071049112 5-7 5-5

424.98605300704855 5-7 5-6

166.79848741240167 5-7 5-7

454.4468138118923 5-7 5-8

-15.522764701306432 5-7 5-9

-1.2867854337624388e-08 5-8 5-0

6.457967325346649e-07 5-8 5-1

-2.3340609734967178e-05 5-8 5-2

0.000708973376162272 5-8 5-3

-0.019438631267507653 5-8 5-4

0.5087714734517893 5-8 5-5

-13.682719341271302 5-8 5-6

454.4468138118923 5-8 5-7

177.83288004456205 5-8 5-8

482.1384799253084 5-8 5-9

8.465919077009912e-10 5-9 5-0

-3.8897014775624105e-08 5-9 5-1

1.3720165772402965e-06 5-9 5-2

-4.0488747061298784e-05 5-9 5-3

0.0010648203194625792 5-9 5-4

-0.026106447381160747 5-9 5-5

0.6235968964734734 5-9 5-6

-15.52276470130644 5-9 5-7

482.1384799253084 5-9 5-8

188.88067260506608 5-9 5-9

41.158674803481624 6-0 6-0

166.9028837596028 6-0 6-1

-2.8640250882142624 6-0 6-2

0.0803289134152152 6-0 6-3

-0.0029296463038361864 6-0 6-4

0.00012755028233213395 6-0 6-5

-6.343126138119715e-06 6-0 6-6

3.507595103258156e-07 6-0 6-7

-2.1246453152378692e-08 6-0 6-8

1.4507310908149777e-09 6-0 6-9

166.9028837596028 6-1 6-0

53.31569391722762 6-1 6-1

236.10575738892214 6-1 6-2

-4.963551573602373 6-1 6-3

0.1607991172255785 6-1 6-4

-0.006558550881877068 6-1 6-5

0.0003128885614382959 6-1 6-6

-1.6811951609518868e-05 6-1 6-7

9.944116947657805e-07 6-1 6-8

-6.372066851942651e-08 6-1 6-9

-2.864025088214266 6-2 6-0

236.10575738892214 6-2 6-1

65.4894273637104 6-2 6-2

289.2544318047409 6-2 6-3

-7.02364871315266 6-2 6-4

0.25446945831484324 6-2 6-5

-0.01137304052363941 6-2 6-6

0.0005862157496450138 6-2 6-7

-3.368244399482017e-05 6-2 6-8

2.113335732190292e-06 6-2 6-9

0.08032891341521697 6-3 6-0

-4.96355157360237 6-3 6-1

289.2544318047409 6-3 6-2

77.67991743137193 6-3 6-3

334.1006192884814 6-3 6-4

-9.072825450457955 6-3 6-5

0.360191033843706 6-3 6-6

-0.0173929530158059 6-3 6-7

0.000958683573508257 6-3 6-8

-5.844190811243732e-05 6-3 6-9

-0.0029296463038361864 6-4 6-0

0.1607991172255785 6-4 6-1

-7.02364871315266 6-4 6-2

334.1006192884814 6-4 6-3

89.88720660115014 6-4 6-4

373.64592803547606 6-4 6-5

-11.118436921248858 6-4 6-6

0.47690775477216274 6-4 6-7

-0.024626177194360107 6-4 6-8

0.0014401268854102951 6-4 6-9

0.00012755028233257804 6-5 6-0

-0.006558550881877068 6-5 6-1

0.2544694583148468 6-5 6-2

-9.07282545045797 6-5 6-3

373.64592803547606 6-5 6-4

102.11133746348824 6-5 6-5

409.42930042601745 6-5 6-6

-13.163259981459504 6-5 6-7

0.6037777027957851 6-5 6-8

-0.03307822996863226 6-5 6-9

-6.343126137675625e-06 6-6 6-0

0.00031288856143651955 6-6 6-1

-0.011373040523645628 6-6 6-2

0.36019103384370954 6-6 6-3

-11.118436921248858 6-6 6-4

409.42930042601745 6-6 6-5

114.35235282121945 6-6 6-6

442.36466857992434 6-6 6-7

-15.208580658635768 6-6 6-8

0.7401259447701689 6-6 6-9

3.507595103258156e-07 6-7 6-0

-1.681195161440385e-05 6-7 6-1

0.0005862157496476783 6-7 6-2

-0.017392953015800572 6-7 6-3

0.4769077547721574 6-7 6-4

-13.163259981459532 6-7 6-5

442.36466857992434 6-7 6-6

126.61029562957819 6-7 6-7

473.0473361389812 6-7 6-8

-17.25507817212126 6-7 6-9

-2.1246454262601717e-08 6-8 6-0

9.944116947657805e-07 6-8 6-1

-3.3682443993043815e-05 6-8 6-2

0.0009586835735033721 6-8 6-3

-0.024626177194370765 6-8 6-4

0.6037777027957869 6-8 6-5

-15.208580658635775 6-8 6-6

473.0473361389812 6-8 6-7

138.88520888562502 6-8 6-8

501.89068297028183 6-8 6-9

1.450730202636558e-09 6-9 6-0

-6.372066763124809e-08 6-9 6-1

2.1133357268612215e-06 6-9 6-2

-5.844190811643412e-05 6-9 6-3

0.0014401268854085188 6-9 6-4

-0.03307822996863226 6-9 6-5

0.740125944770176 6-9 6-6

-17.25507817212126 6-9 6-7

501.89068297028183 6-9 6-8

151.17713556953166 6-9 6-9
